# Biomarkers of hypoxic–ischemic encephalopathy: a systematic review

**DOI:** 10.1007/s12519-023-00698-7

**Published:** 2023-04-21

**Authors:** Inês Caramelo, Margarida Coelho, Miguel Rosado, Carla M. P. Cardoso, Alexandra Dinis, Carlos B. Duarte, Mário Grãos, Bruno Manadas

**Affiliations:** 1 CNC-Center for Neuroscience and Cell Biology, University of Coimbra, 3004-504 Coimbra, Portugal; 2 PhD Programme in Experimental Biology and Biomedicine, Institute for Interdisciplinary Research (IIIUC), University of Coimbra, Casa Costa Alemão, 3030-789 Coimbra, Portugal; 3 Chemistry Department, Faculty of Sciences and Technology, University of Coimbra, 3004-535 Coimbra, Portugal; 4 Stemlab SA, 3060-197 Cantanhede, Portugal; 5 Pediatric Intensive Care Unit, Hospital Pediátrico, Centro Hospitalar E Universitário de Coimbra, 3000-075 Coimbra, Portugal; 6 Department of Life Sciences, University of Coimbra, 3001-401 Coimbra, Portugal; 7 Biocant, Technology Transfer Association, 3060-197 Cantanhede, Portugal; 8 Institute for Interdisciplinary Research, University of Coimbra (IIIUC), 3030-789 Coimbra, Portugal

**Keywords:** Biomarker, Hypoxic–ischemic encephalopathy, Neonatal brain injury, Newborn, Neuron-specific enolase, S100-calcium-binding protein-B

## Abstract

**Background:**

Current diagnostic criteria for hypoxic–ischemic encephalopathy in the early hours lack objective measurement tools. Therefore, this systematic review aims to identify putative molecules that can be used in diagnosis in daily clinical practice (PROSPERO ID: CRD42021272610).

**Data sources:**

Searches were performed in PubMed, Web of Science, and Science Direct databases until November 2020. English original papers analyzing samples from newborns > 36 weeks that met at least two American College of Obstetricians and Gynecologists diagnostic criteria and/or imaging evidence of cerebral damage were included. Bias was assessed by the Newcastle–Ottawa Scale. The search and data extraction were verified by two authors separately.

**Results:**

From 373 papers, 30 met the inclusion criteria. Data from samples collected in the first 72 hours were extracted, and increased serum levels of neuron-specific enolase and S100-calcium-binding protein-B were associated with a worse prognosis in newborns that suffered an episode of perinatal asphyxia. In addition, the levels of glial fibrillary acidic protein, ubiquitin carboxyl terminal hydrolase isozyme-L1, glutamic pyruvic transaminase-2, lactate, and glucose were elevated in newborns diagnosed with hypoxic–ischemic encephalopathy. Moreover, pathway analysis revealed insulin-like growth factor signaling and alanine, aspartate and glutamate metabolism to be involved in the early molecular response to insult.

**Conclusions:**

Neuron-specific enolase and S100-calcium-binding protein-B are potential biomarkers, since they are correlated with an unfavorable outcome of hypoxic–ischemic encephalopathy newborns. However, more studies are required to determine the sensitivity and specificity of this approach to be validated for clinical practice.

**Supplementary Information:**

The online version contains supplementary material available at 10.1007/s12519-023-00698-7.

## Introduction

Perinatal asphyxia (PA) can lead to severe brain injury and is the most frequent cause of hypoxic–ischemic encephalopathy (HIE), occurring in 1–8/1000 live births [1]. Several conditions might lead to an interruption of the blood flow to the brain, resulting in an insufficient supply of oxygen and nutrients to the brain required to maintain the high energy demands of this organ [2]. Briefly, after the initial insult, energy failure results in an impairment of active membrane transport and, consequently, membrane depolarization and glutamate release. Its accumulation in the synaptic cleft leads to increased excitotoxicity, culminating in cytotoxic edema, activation of inflammatory and apoptotic pathways, and finally neuronal death [3]. These events may lead to permanent sequelae in the neonatal brain, namely, epilepsy, cerebral palsy, mental disability, motor and sensorial impairment, or even death [4].

There are no accurate and objective tools with high sensitivity and specificity to diagnose newborns suffering from HIE. Some complementary blood tests have been proposed to evaluate liver and renal function and support the diagnosis, but they lack neuronal specificity [5]. According to the American College of Obstetricians and Gynecologists (ACOG) [6], if more than one of the following criteria is met, the newborn is more likely to suffer from a peripartum hypoxic–ischemic event: (1) appearance, pulse, grimace, activity, respiration (APGAR) score below 5 at 5 and 10 minutes; (2) fetal umbilical artery pH less than 7.0 and/or base deficit equal to or greater than 12 mmol/L; (3) neuroimaging evidence of acute brain injury seen on magnetic resonance imaging (MRI) or magnetic resonance spectroscopy (MRS), and (4) presence of multiorgan failure. However, imaging is usually performed in the first two weeks of life and is not suitable for a rapid diagnosis [5].

The therapeutic window to treat HIE is limited to the first 6 hours of life, before the beginning of inflammatory and apoptotic pathways [7]. Presently, therapeutic hypothermia (TH) is the treatment standard for moderate to severe cases of HIE, consisting of cooling either the newborn’s whole-body temperature (keeping it between 32 °C and 34 °C) or selectively the head for up to 72 hours. This approach aims to slow the metabolic rate and the accumulation of inflammatory cytokines, lowering the activation of intracellular pathways leading to programmed cell death. Furthermore, innovative treatments are emerging, including drugs, such as topiramate, erythropoietin, and stem cells, which are not yet used as standard guideline treatments [8]. The lack of a definitive test to diagnose HIE might lead to a misdiagnosis and a lack of proper treatment choices that can have an irretrievable impact on these neonates’ future.

Several studies have been published in recent decades proposing hypothetical biomarkers for HIE [5, 9]. Nevertheless, to our knowledge, there is no review on the literature that collects all these data. Therefore, this systematic review proposes to critically assess potential biomarkers for the diagnosis of term newborns who have been diagnosed with HIE in accordance with ACOG criteria and/or MRI brain injury evidence.

## Methods

The study design was registered on PROSPERO on 1st October 2021 (ID: CRD42021272610) [10]. In addition, this review was written in accordance with PRISMA guidelines [11]. The search strategy, study eligibility, and quality assessment were performed by IC and MC, while IC and MR performed data extraction. The evaluation was performed independently, and disagreements were resolved by consensus.

### Search strategy

An article search was conducted in three distinct databases until November 11, 2020: PubMed, Web of Science, Science Direct, and OpenGrey. Since HIE terminology is not consensual, four different terms were used: “neonatal brain injury”, “neonatal encephalopathy”, “hypoxic–ischemic encephalopathy” and “neonatal hypoxic–ischemic encephalopathy”. These terms were combined with the preposition and with the terms “biomarker*”, “proteomic*”, “metabolomic*”, restricting it to the title and abstract fields. The methods are described in detail in the supplementary material.

### Study eligibility

Selected articles were subjected to abstract evaluation and three selection phases. The first approach aimed to categorize the results by document type, language, species of the samples studied, and biomarker type. Only original English papers in which the research was focused on biochemical HIE biomarkers in human samples were selected for method evaluation. The studies were then classified according to study type, sample size, sample type, association with other pathologies, gestational age (GA) or age, disease, sample collection time, therapy, outcome assessment, and association of the biomarkers with multiorgan failure. Only studies regarding term newborns collected in the first 72 hours of life were selected for a diagnostic criteria analysis. Moreover, studies analyzing cerebrospinal fluid (CSF) were excluded, since its collection from newborns may be considered unethical in many countries.

Since diagnosis criteria for HIE are not standardized in all studies, to analyze a homogenous population, the diagnostic criteria applied were assessed in each study: APGAR score, fetal acidemia, MRI and multiorgan failure. Studies that matched at least two ACOG diagnostic criteria or had neuroimaging evidence of brain injury were selected for quality assessment and data extraction.

### Quality assessment

The quality of each study was evaluated by the Newcastle–Ottawa Scale (NOS) [12]. In accordance with the authors’ guidelines, different scales were applied depending on the study type (cohort or case–control). The scores for selection, comparability, and outcomes are presented separately.

### Data extraction

Population characteristics were analyzed to infer the homogeneity of the populations being reviewed in this manuscript. Information about the study location, type of study, gestational age and/or birth weight, diagnosis criteria, HIE severity assessment, complementary diagnostic exams, therapeutic hypothermia, sample size, sample type, and the biomarker described in the study was extracted. In addition, information about the biomarker, namely, the biomarker type, the technique used to analyze the biomarker, the sample size of each group, collection time, and *P* value (when available), was also extracted. The extracted data is available in Tables 1-3 and in the supplementary material.

### Data analysis

Venn diagrams were generated on InteractiVenn [13]. A UniProt accession number, Human Metabolome Database (HMBD) or GeneCard code was manually attributed to identified proteins, metabolites, or genes, respectively. Proteins and metabolites identified in serum and plasma samples were subjected to further analysis. Protein gene ontology (GO) analysis was performed on the DAVID Bioinformatic Database [14], and images were generated using the ggplot2 R package [15]. Metabolite pathway and mixomic analyses were performed on MetaboAnalyst [16].

## Results

### Literature search and study selection

**Fig. 1:**
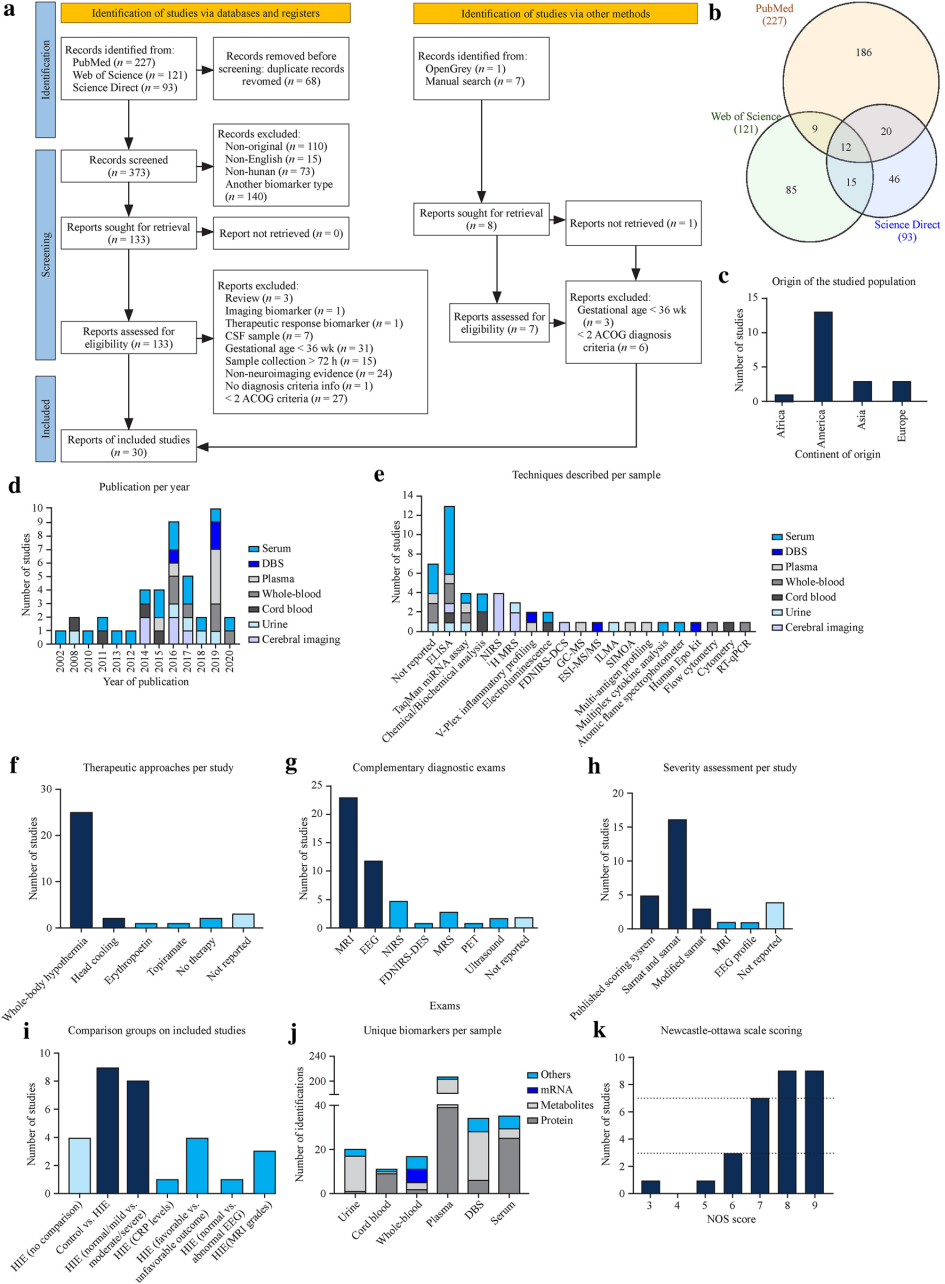
Database search results and summary of population characteristics. **a** PRISMA 2020 flow diagram. Only English manuscripts that analyzed human samples collected within 72 h (excluding CSF) and studied biochemical biomarkers associated with HIE were included. In addition, selected studies had to match at least two ACOG diagnosis criteria or present neuroimaging evidence of acute brain ischemia. **b** Common articles between PubMed, Web of Science, and Science Direct. Only 12 articles were common between the three databases. **c** and **d** Concerning population characteristics, the majority of selected studies were performed in Europe and America and published after 2014. **f**, **g** Almost all studies applied whole-body hypothermia and had MRI data available. **h** Studies lacked uniformity in comparison groups, but the severity was preferably assessed by the Sarnat scoring system. **e**, **j** Technique most prevalently used is ELISA, while plasma was the fluid with more identifications. **k** Summary of NOS scoring. *CSF* cerebrospinal fluid, *ACOG* American College of Obstetricians and Gynecologists, *DBS* dried blood spots, *ELISA* enzyme linked immunosorbent assay, *miRNA* microRNA, *NIRS* near-infrared spectroscopy, *MRS* magnetic resonance spectroscopy, *FDNIRS–DCS* frequency-domain near-infrared spectroscopy–diffuse correlation spectroscopy, *MS* mass spectrometer, *GC–MS* gas chromatography–mass spectrometer, *ESI–MS* electro spray ionization MS, ILMA immunoluminometric assay, *SIMOA* single molecular array, *Epo* erythropoietin, *RT–PCR* reverse transcription–polymerase chain reaction, *MRI* magnetic resonance imaging, *EEG* electroencephalogram, *PET* positron emission computed tomography, *CRP* C-reactive protein, *NOS* Newcastle–Ottawa scale

The PubMed search resulted in 279 hits and 227 unique records. The Web of Science search resulted in 233 hits and 121 unique records. The Science Direct search resulted in 145 hits and 93 unique records. After duplicate removal, a list of 373 unique records was obtained (Fig. 1a, b). From these, a total of 240 records were excluded considering nonoriginal records (*n* = 110), non-English records (*n* = 15), nonhuman studies (*n* = 73) and nonbiochemical, therapeutic response, genetic, pH, or associated with other disease biomarker studies (*n* = 140), resulting in 133 records for evaluation of the methods. During this selection phase, three articles were reclassified as review articles, one was reclassified as an imaging biomarker article, and four focused on therapeutic response biomarkers. Then, studies analyzing CSF samples were excluded (*n* = 7), because the collection of this fluid is not a common clinical practice in newborns. Works studying children, adults, and newborns < 36 week GA were also excluded (*n* = 31), since this review focuses on HIE biomarkers for term newborns. Finally, articles in which sample collection was not within the first 72 hours after birth (*n* = 15) were excluded, as this review focuses on identifying biomarkers of HIE to be used as an early diagnostic tool. These steps identified 81 records, of which diagnostic criteria were analyzed. It was not possible to obtain diagnosis criteria information in one of the reports; therefore, it was excluded. Studies that matched at least two ACOG diagnostic criteria or had neuroimaging evidence of acute brain injury by MRI or MRS (*n* = 29) were selected. Regarding the manual search, one study matched all eligibility criteria and was considered for further analysis. In conclusion, 30 studies were included in this systematic review [17–46] (Fig. 1a).

### Quality assessment

Considering the NOS, no case–control study was considered to be at risk of bias (Supplementary Tables 1–2). Regarding cohort studies, one article was considered to be at high risk of bias (score ≤ 3), and four were at medium risk (score < 7). Five articles were identified as being potentially biased (Fig. 1k). These articles were not excluded from further analysis.

### Studies and population characteristics

The population characteristics are summarized in Table 1, and the studies included were published between 2002 and 2020, despite the majority being released after 2014 (Fig. 1d). In addition, most studies took place in Europe and America (Fig. 1c) and are prospective studies. Regarding population characteristics, all studies included newborns older than 36 weeks of gestation that matched at least two ACOG diagnosis criteria or neuroimaging evidence of brain injury to allow studying a more homogeneous population. Moreover, almost all studies used whole-body hypothermia as a therapeutic approach and MRI as a complementary exam (Fig. 1f, g).

**Table 1:** Summary of studies and population characteristics

References		Year of publication	Study location	Study type	Gestational age/birth weight	Diagnosis criteria	Severity evaluation	Complementary exams	Therapeutic approaches	Sample size	Sample type	Biomarker
Akamatsu, Sugiyama et al. 2019	[19]	2019	Tokyo, Japan	Prospective	≥ 36 wk and birth weight ≥ 1800 g	(1) APGAR score ≤ 5 at 10 min; (2) pH ≤ 7 or base deficit ≥ 16 mmol/L in any blood within 1 h of birth; (3) need for resuscitation or assisted ventilation ≥ 10 min after birth	Modified Sarnat	MRI within 3–4 weeks	Whole-body hypothermia	78	Plasma	sLOX-1
Alshweki, Perez-Munuzuri et al. 2017	[35]	2017	Santiago de Compostela, Spain	Prospective	≥ 36 wk and birth weight ≥ 1800 g	(1) APGAR score < 5 at 5 min; (2) pH ≤ 7 on arterial cord blood; (3) need for prolonged major resuscitation	Sarnat and Sarnat	MRI; EEG; PET	Whole-body hypothermia	31	Serum	NSE, S100B
											Urine	S100B
Balada, Tebe et al. 2020	[20]	2020	Barcelona, Spain	Prospective	≥ 36 wk and birth weight ≥ 1800 g	(1) APGAR score ≤ 5 at 10 min; (2) pH ≤ 7 or base deficit ≥ 16 mmol/L in umbilical cord blood or arterial, venous or capillary blood within 1 h of birth; (3) need for resuscitation for more than 10 min after birth; (4) neurological dysfunction manifested by subnormal level of unconsciousness with or without seizures or palmary hyperexcitability, tremor, overactive myotatic reflexes, hypersensitivity to stimulation or startle responses	Scoring system available on supplementary data	MRI; EEG; MODs (3 d); GMFCS, BFMF and BSITD-III at 24 mon	Whole-body hypothermia	58	Whole blood	MMP9; IL-8; HSPA1A; CCR5; PPARG; TLR8
Bale, Mitra et al. 2014	[41]	2014	London, England	Prospective	≥ 36 wk	(1) pH ≤ 7 or base deficit ≥ 16 mmol/L in cord blood any blood sample in 1 h; (2) APGAR score ≤ 5 at 10 min; (3) need for resuscitation or mask ventilation at 10 min	Sarnat and Sarnat	MRI (up to 7 d); EEG; NIRS; ^1^H MRS	Whole-body hypothermia	6	Cerebral imaging	ΔSpO_2_ (systemic oxygen saturation), Δ[HbD] (= oxygenated ‒ deoxygenated haemoglobin), Δ[HbT] (= oxygenated + deoxygenated haemoglobin), Δ[oxCCO] (oxidation state of cytochrome-c oxidase), Lac/NAA ratio
Bersani, Ferrari et al. 2019	[44]	2019	Alessandria, Modena, Rome, Italy; Warsaw, Poland; Cairo, Egypt	Retrospective case–control	> 36 wk	(1) pH < 7 in umbilical artery or base excess ≤ − 12 mmol/L in cord blood or venous blood within 1 h; (2) APGAR score < 3 at 5 min; (3) occurrence of multiorgan failure; (4) need for resuscitation and/or positive pressure ventilation for more than 3 min	Sarnat and Sarnat	EEG; neurological examination by Prechtl	Whole-body hypothermia and no therapy	108	Plasma	Glucose
											Whole blood	Creatinine; Urea
											Urine	S100B
Chalak, Sánchez et al. 2014	[18]	2014	Dallas, Texas, USA	Prospective	≥ 36 wk and birth weight ≥ 1800 g	(1) pH ≤ 7 or base deficit ≥ 16 mEq/L in umbilical arterial cord plasma; (2) if history of acute perinatal event or blood pH between 7.01–7.15 or base deficit between 10–15.9 mmol/L: combination with (a) APGAR score ≤ 5 at 10 min or (b) need for assisted ventilation at 10 min after birth	Sarnat and Sarnat	MRI at 7–14 d; BSID-III at 15–18 mon	Whole-body hypothermia	27	Umbilical cord arterial serum	GFAP; UCHL1; IL-1; IL-6; IL-8; VEGF; IFN-γ; TNF, RANTES
											Umbilical cord arterial plasma	GFAP; UCHL1
Chouthai, Sobczak et al. 2015	[46]	2015	Michigan, USA	Retrospective case–control	≥ 36 wk	(1) base deficit ≥ 16 mmol/L or pH ≤ 7.0 in cord blood or any blood sample, within the first hour of life; (2) if blood analysis revealed pH between 7.01–7.15 or base deficit between 10–15.9 mmol/L or not available, additional criteria requested (a) existence of perinatal data compatible with perinatal asphyxia, as cord prolapse or (b) APGAR score ≤ 5 at 10 min or need for assisted ventilation at 10 min after birth	Moderate/severe disability criteria (https://doi.org/10.1056/NEJMcps050929)	MRI	Whole-body hypothermia or no therapy	56	Serum	Glucose
Dehaes, Aggarwal et al. 2014	[30]	2014	Massachusetts, USA	Prospective	≥ 36 wk and birth weight ≥ 2000 g	(1) APGAR score ≤ 5 at 10 min; (2) pH ≤ 7 or base deficit ≥ 16 mEq/L within 1 h of birth; (3) evidence of neonatal encephalopathy by physical exam; (4) abnormal cerebral function monitor evidenced by seizures or amplitude-EEG; (5) need for ventilation for at least 10 min after birth	–	MRI within 6 d; FDNIRS–DCS; GMFCS and MDI at 18 mon	Whole-body hypothermia	27	Cerebral imaging	CMRO_2_; CBF; CBV; SO_2_
Douglas-Escobar, Yang et al. 2010	[22]	2010	Florida, USA	Prospective	≥ 38 wk	(1) APGAR score < 3 at 5 min; (2) presence of multiorgan failure; (3); neurological symptoms, as seizures, coma or hypotonia	Sarnat and Sarnat	MRI (1 to 5 d)	Whole-body hypothermia	28	Serum	pNF-H; UCHL1
El-Mazary, Abdel-Aziz et al. 2015	[40]	2015	Minia, Egypt	Prospective	≥ 37 wk	(1) pH ≤ 7 in umbilical artery or base deficit ≥ 16 mmol/L; (2) APGAR score ≤ 3 for more than 10 min; (3) disturbed conscious level, abnormal neuromuscular control or abnormalities in complex reflexes or autonomic function; (4) presence of seizures	Sarnat and Sarnat	–	–	80	Serum	Selenium, sodium, potassium, calcium, urea, creatinine, hemoglobin, ALT, AST
											Whole blood	Platelet: WBC
Ennen, Huisman et al. 2011	[17]	2011	Baltimore, USA	Prospective	≥ 36 wk	(1) pH < 7 in umbilical artery or base deficit > 12 mM; (2) APGAR score < 7 at 5 min	Sarnat and Sarnat	MRI	Whole-body hypothermia	46	Cord blood	GFAP
											Serum	GFAP
Ezgu, Atalay et al. 2002	[39]	2002	Ankara, Turkey	Prospective	≥ 37 wk	(1) pH ≤ 7.2 in cord blood; (2) APGAR score ≤ 6 at 5 min	Sarnat and Sarnat	MRI (up to 3 d); EEG Griffiths’ developmental scales (1 y)	–	26	Serum	NSE
											CSF	NSE
Fredly, Nygaard et al. 2016	[37]	2016	Oslo, Norway	Prospective	≥ 36 wk	(1) an Apgar score ≤ 5 at 10 min; (2) a need for respiratory support at 10 min following birth (3) pH ≤ 7.00 or a base deficit ≥ 16 mmol/L obtained via either umbilical arterial blood or any blood samples taken within 60 min of birth	Sarnat and Sarnat	MRI; NIRS	Whole-body hypothermia	28	Serum	Lactate; CRP; cerebral oxygenation
Haiju, Suyuan et al. 2008	[45]	2008	Shandong, China	Prospective	≥ 37 wk	(1) APGAR < 3 at 5 min; (2) pH < 7.0 in first arterial blood; (3) presence of encephalopathy at least one: hypotonia, abnormal reflexes, absent or weak suck, or clinical seizures; (4) multiple organ failure	Sarnat and Sarnat	EEG (in case of seizures); neuroimaging; BSID-II at 4, 8, 12, 18–24 mon	–	63	Cord blood	NRBC/per 100WBC; lactate
Jain, Pagano et al. 2017	[27]	2017	St. Louis, Missouri, and Nashville, Tennessee, USA	Prospective	> 36 wk	(1) APGAR < 5 at 10 min; (2) base deficit > 16 mmol/L or pH < 7.0 in cord blood or any blood sample within the first hour of life; (3) requiring assisted ventilation for at least 10 min	Sarnat	NIRS (for 48 h); MRI (second week); BSID at 18 to 24 mon	Whole-body hypothermia; Head cooling (*n* = 7)	21	Cerebral imaging	CrSO_2_
Jones, Heep et al. 2018	[34]	2017	North Bristol, England	Retrospective	≥ 36 wk	(1) APGAR ≤ 5 at 10 min; (2) base deficit > 16 mmol/L or pH < 7.0 in cord blood or any blood sample within first hour of life; (3) requiring assisted ventilation at 10 min of life	EEG profile	EEG	Whole-body hypothermia	79	Serum	Lactate, Glucose, Troponin T, CK, LDH, ALT, AST, GGT, CRP, Alk Phos
Locci, Noto et al. 2018	[43]	2018	Cuneo, Italy	Prospective	≥ 36 wk and birth weight ≥ 2500 g	(1) APGAR ≤ 5 at 10 min; (2) base deficit ≥ 12 mmol/L or pH < 7.0 in cord blood or any blood sample within first hour of life; (3) required resuscitation at 10 min	Sarnat	MRI (1 wk–1 mon); EEG; Head ultrasound (130 d)	Whole-body hypothermia	26	Urine	Lactate, myoinositol, betaine, taurine, arginine, acetate, N-Ac-Groups, pyruvate, succinate, glutamine, acetone, citrate, DMA, α-ketoglutarate
Lopez-Suarez, Concheiro-Guisan et al. 2019	[21]	2019	Galicia, Spain	Retrospective	≥ 37 wk and birth weight ≥ 2500 g	(1) pH ≤ 7.0 in cord blood; (2) APGAR score < 5 at 5 min; (3) prolonged major resuscitation; (4) sentinel events of fetal distress	Sarnat	MRI; EEG	Whole-body hypothermia	894	Serum	NSE
											DBS	Acylcarnitine profile
Maggiotto, Sondhi et al. 2019	[29]	2019	California, San Francisco, USA	Prospective	> 36 wk and birth weight > 2000 g	(1) base deficit > 12 mEq/L or pH ≤ 7.0 in cord blood gas or any blood sample in the first hour of life; (2) APGAR score ≤ 5 at 10 min; (3) abnormal neurological examination; (4) seizures	Neurological score	MRS and MRI at 4–13 d; EEG; BSID-III at 6, 12, 18 and/or 24 mon	Whole-body hypothermia	19	Whole blood	RBC(GLUT1); WBC(GLUT3);
											Plasma	NSE; GFAP
Massaro, Chang et al. 2014	[36]	2014	Washington DC, USA	Prospective	> 36 wk and > 1800 g	(1) signs of moderate or severe encephalopathy by EEG; (2) base deficit > 16 mmol/L or pH < 7.0 in cord blood or any blood sample in the first hour of life	modified Sarnat	EEG; BSID-II at 15 mon	Whole-body hypothermia	83	Serum	NSE; S100B
Massaro, Chang et al. 2012	[28]	2012	California, San Francisco, USA (multicentered)	Prospective	> 36 wk and > 1800 g	(1) base deficit ≥ 16 mmol/L or pH ≤ 7.0 in cord blood or any blood sample, within the first hour of life; (2) if blood analysis revealed pH between 7.01–7.15 or base deficit between 10–15.9 mmol/L or not available, additional criteria requested (a) existence of perinatal data compatible with perinatal asphyxia, as cord prolapse or (b) APGAR score ≤ 5 at 10 min or need for assisted ventilation at 10 min after birth	MRI (basal ganglia score and cortical/watershed score)	MRI (7–10 d); Amiel-Tison neurological assessment (14th day)	Whole-body hypothermia	75	Serum	NSE; S100B
Massaro, Jeromin et al. 2013	[25]	2013	California, San Francisco, (multicentered)	Prospective	> 36 wk and > 1800 g	(1) base deficit ≥ 16 mmol/L or pH ≤ 7.0 in cord blood or any blood sample, within the first hour of life; (2) if blood analysis revealed pH between 7.01–7.15 or base deficit between 10–15.9 mmol/L or not available, additional criteria requested (a) existence of perinatal data compatible with perinatal asphyxia, as cord prolapse or (b) APGAR score ≤ 5 at 10 min or need for assisted ventilation at 10 min after birth	Moderate/severe disability (https://doi.org/10.1056/NEJMcps050929)	NIRS; EEG from admission to 12 h after rewarming; MRI at 5–14 d of life	Whole-body hypothermia	20	Serum	UCHL1; GFAP
Massaro, Wu et al. 2019	[32]	2019	California, San Francisco (multicentered)	Prospective	≥ 36 wk and birth weight ≥ 1800 g	(1) APGAR ≤ 5 at 10 min; (2) base deficit ≥ 15 mmol/L or pH < 7.0 in cord blood or any blood sample within the first hour of life; (3) required resuscitation at 10 min	Modified Sarnat	MRI 4–7 d of age; AIMS and WIDEA at 12 mon	Whole-body hypothermia or head cooling + erythropoietin	50	DBS	S100B; TNF-α; IL-1β; IL-6; IL-8; Epo
											Plasma	S100B; TNF-α; IL-1β; IL-6; IL-8; Epo
Mitra, Bale et al. 2016	[26]	2016	London, England	Prospective	≥ 36 wk	MRI	–	NIRS; ^1^H MRS	Whole-body hypothermia	14	Perri cerebral imaging	Δ oxCCO; ΔHbD
												Lac/NAA
Oh, Perritt et al. 2008	[23]	2008	USA (multicentered)	Prospective	≥ 36 wk and > 2000 g	(1) base deficit ≥ 16 mmol/L or pH ≤ 7.0 in cord blood or any blood sample, within first hour of life; (2) if blood analysis revealed pH between 7.01 and 7.15 or base deficit between 10–15.9 mmol/L or not available, additional criteria requested (a) existence of perinatal data compatible with perinatal asphyxia, as cord prolapse or (b) APGAR score ≤ 5 at 10 min or need for assisted ventilation at 10 min after birth	Moderate/severe disability criteria (https://doi.org/10.1056/NEJMcps050929)	–	Whole-body hypothermia	58	Urine	Lactate/creatinine ratio
Pineiro-Ramos, Nunez-Ramiro et al. 2020	[24]	2020	Valencia, Spain (multicentered)	Prospective	≥ 36 wk and > 2000 g	(1) base deficit ≥ 16 mmol/L and/or pH ≤ 7.0 in cord blood or any blood sample, within the first hour of life; (2) if blood analysis revealed pH between 7.01–7.15 or base deficit between 10 and 15.9 mmol/L or not available, additional criteria requested (a) existence of perinatal data compatible with perinatal asphyxia, as cord prolapse or (b) APGAR score ≤ 5 at 10 min or need for assisted ventilation at 10 min after birth	Sarnat and Sarnat	MRI 4–8 d after birth	Whole-body hypothermia + topiramate/placebo	62	Plasma	Pyruvate; lactate
												Alanine, aspartate, and glutamate metabolism; arginine and proline metabolism; caffeine metabolism; D-glutamine and D-glutamate metabolism; limonene and pinene degradation; lysine biosynthesis; lysine degradation; nitrogen metabolism; phenylalanine metabolism; seleno amino acid metabolism; steroid hormone biosynthesis
Ponnusamy, Kapellou et al. 2016	[31]	2016	London, England	Prospective	≥ 36 wk and ≥ 2000 g	(1) APGAR ≤ 5 at 10 min; (2) continued need for resuscitation at 10 min after birth; (3) base deficit ≥ 16 mmol/L or pH ≤ 7.0 in cord blood or any blood sample, within the first hour of life	–	MRI 5–35 d of life	Whole-body hypothermia	30	Plasma	RNU6B; Let7b; miR-21
											EDTA–blood	RNU6B; Let7b; miR-21
											Urine	RNU6B; Let7b; miR-21
											DBS	RNU6B; Let7b; miR-21; miR-29b; miR-124; miR-155
Saito, Shibasaki et al. 2016	[33]	2016	Yokohama, Japan	Prospective	≥ 36 wk and ≥ 2000 g	(1) APGAR ≤ 5 at 10 min; (2) continued need for resuscitation at 10 min after birth; (3) base deficit ≥ 16 mmol/L or pH ≤ 7.0 in cord blood or any blood sample, within the first hour of life	Sarnat and Sarnat	MRI	Whole-body cooling	22	Serum	CRP; IL-6; PCT
											Whole blood	WBCC; neutrophil count; platelet count
Shaikh, Boudes et al. 2015	[42]	2015	Monteral, Canada	Prospective	≥ 36 wk and ≥ 2000 g	(1) base deficit ≥ 16 mmEq/L or pH ≤ 7.0 in postnatal blood within the first hour of life; (2) APGAR score ≤ at 5 and 10 min; (3) need for ventilation at least 10 min; (4) evidence of moderate/severe NE by neurological exam or EEG	–	MRI 9–13 d of life	Whole-body hypothermia	16	Plasma	Ang-2; Cathepsin D; Hepsin; MMP-3; MMP-7; MMP-9; MMP-10; Ang; AXL; Endoglin; EGFR; FABP-4; Galectin-3; G6PI; HB-EGF; HER-2; IGFBP-2; IGFBP-3; ICAM-1; KLK-5; MSP; NP-1; ErbB3; SCF; SOD-1; TN-C; TNFR2; VEGF-C; VEGFR-2; VEGFR-3; YKL-40; BDNF; NSE; Nr-CAM; cFib; Collagen IV; TIE-2; Endostatin; Fib-1C; HE4; IGFBP-1; IGFBP4; IGFBP5; IGFBP-6; TIMP-1; FasL; FasR; Sortilin; TRAIL-R3
Sweetman, Onwuneme et al. 2017	[38]	2017	Dublin, Ireland	Prospective	≥ 36 wk and ≥ 2000 g	2 of 3: (1) evidence or suspicion of HIE based on the history of fetal distress; (2) need for resuscitation after birth (3) base deficit > 15 mmol/l or pH < 7.2 in cord blood or admission arterial sample	Sarnat and Sarnat	Cranial ultrasound (up to 24 h); MRI (up tp 7 d)	Whole-body hypothermia	94	Serum	VEGF; Epo
										34	CSF	VEGF; Epo

*APGAR* appearance, pulse, grimace, activity, respiration, *EEG* electroencephalogram, *MRI* magnetic resonance imaging, *PET* positron emission computed tomography, *GMFCS* the gross motor function classification system, *BFMF* bimanual fine motor function, *BSITD-III* Bayley scale of infant and toddler development, *CSF* cerebrospinal fluid, *DBS* dried blood spots, *EDTA* ethylene diamine tetraacetic acid, *sLOX-1* soluble lectin-like oxidized low density lipoprotein receptor-1, *NSE* neuron-specific enolase, *S100B* S100-calcium-binding protein-B, *MMP* matrix metalloprotein, *IL* interleukin, *HSPA1A* heat shock protein family A member 1A, *CCR5* C–C motif chemokine receptor 5, *PPARG* peroxisome proliferator activated receptor gamma, *TLR8* toll like receptor 8, *GFAP* glial fibrillary acidic protein, *UCHL1* ubiquitin C-terminal hydrolase L1, *VEGF* vascular endothelial growth factor, *IFN-γ* interferon γ, *TNF* tumor necrosis factor, *RANTES* reduced upon activation, nornal T cell expressed and secreted, *CMRO*_*2*_ cerebral oxygen metabolism index, *CBF* cerebral blood flow index, *CBV* cerebral blood volume, *SO*_*2*_ hemoglobin oxygen saturation, *WBC* white blood cell, *NRBC* nucleated red blood cell, *CRP* C-reactive protein, *CrSO*_*2*_ cerebral regional oxygen saturation, *CK* creatine kinase, *LDH* lactate dehydrogenase, *ALT* alanine aminotransferase, *AST* aspartate aminotransferase, *GGT* γ-glutamyl transpeptidase, *Alk Phos* alkaline phosphatase, *DMA* dynamic muscle activation, *GLUT* glucose transporter, *Epo* erythropoietin, *RNU6B* U6 small nuclear 2, *PCT* procalcitonin, *Ang* angiotensin, *EGFR* epidermal growth factor receptor, *FABP* fatty acid-binding protein, *G6PI* glucose-6-phosphate isomerase, *HB-EGF* heparin-binding epidermal growth factor, *HER-2* human epidermal growth factor receptor 2, *IGFBP* insulin-like growth factor binding protein, *ICAM-1* intercellular cell adhesion molecule-1, *KLK-5* kallikrein 5, *MSP* macrophage stimulating protein, *NP-1* alpha-defensin-1, *SCF* Stem cell factor, *SOD-1* Superoxide dismutase, TN-C Tenascin-C, *TNFR2* tumor necrosis factor receptor-2, *VEGFR* VEGF receptor, *YKL-40* chitinase-3-like protein 1, *BDNF* brain-derived neurotrophic factor, *Nr-CAM* neuronal cell adhesion molecule, cFib Cellular Fibronectin, *TIE-2* tyrosine–protein kinase receptor, Fib-1C Fibulin-1C, *HE4* human epididymis protein 4, *TIMP-1* tissue inhibitor of metalloproteinase-1, *FasL* Fas Ligand, *FasR* Fas receptor, TRAIL-R3 tumor necrosis factor-related apoptosis-inducing ligand receptor 3

Potential biomarkers were identified in the cord blood, plasma, serum, whole blood, dried blood spots (DBS), and urine (Tables 2, 3 and Supplementary Tables 4–7). Interestingly, some studies used advanced cerebral imaging techniques to address the behavior of specific molecules during TH, while enzyme linked immunosorbent assay (ELISA) was the most commonly used technique (Fig. 1e). Although metabolites have a high number of identifications (due to the use of high throughput techniques) (Fig. 1j), most studies have focused their attention on proteins. Nevertheless, recent reports indicate that RNA and microRNA (miRNA) are emerging as possible diagnostic targets.

**Table 2:** Summary of findings of biomarkers present in plasma

References			Sample type	Biomarker	Code	Technique	Group	Collection time	Results	Summary	Observations
Proteins											
Akamatsu, Sugiyama et al. 2019		[19]	Plasma	sLOX-1	P78380	ELISA	Control	< 6 h, 24 h, 48 h–96 h, 120 h–216 h			
							Mild HIE				
							Moderate HIE		Increased levels compared to control (*P* < 0.01)	↑ at first 6 h	
									Levels decreased significantly at 48 h–216 h		
							Severe HIE		Increased levels compared to control (*P* < 0.01)	↑↑ at first 6 h	Increased levels of moderate and severe HIE compared to mild HIE (*P* < 0.01)
									Increased levels compared to mild HIE (*P* < 0.05)		
Maggiotto, Sondhi et al. 2019		[29]	Plasma	NSE	P09104	SIMOA	Control	6 h–76 h			
							HIE	Pre-TH; during TH; rewarming; pos-TH	Increased levels at pre-TH compared to control groups (*P* < 0.05)	↑ at pre-TH	
									Levels decreased during, rewarming and pos TH (*P* < 0.01)		
				GFAP	P14136	SIMOA	Control	6 h–76 h			
							HIE	Pre-TH; during TH; rewarming; pos-TH	No significant differences	= on all timepoints	
Massaro, Wu et al. 2019		[32]	Plasma	S100B	P04271	ELISA	HIE	< 24 h, 120 h	No comparisons between groups were performed		
				IL-1β	P01584	V-PLEX proinflammatory panel					
				IL-6	P05231						
				IL-8	P10145						
				TNF-α	P01375						
				Erythropoietin	P01588	Human EPO base kit					
Shaikh, Boudes et al. 2015		[42]	Plasma	MMP-9	P14780	Multi-analyte profiling antigen analysis	Control	24 h			
							HIE	6 h, 24 h, 48 h, 72 h, 96 h	Decreased levels compared to healthy controls (*P* < 0.05)	↓ at 24 h	
				FABP4	P15090		Control	24 h			
							HIE	6 h, 24 h, 48 h, 72 h, 96 h	Increased levels compared to healthy controls (*P* < 0.05)	↑ at 24 h	
				Galectin-3	P17931		Control	24 h			
							HIE	6 h, 24 h, 48 h, 72 h, 96 h	Increased levels compared to healthy controls (*P* < 0.05)	↑ at 24 h	
				KLK-5	Q9Y337		Control	24 h			
							HIE	6 h, 24 h, 48 h, 72 h, 96 h	Decreased levels compared to healthy controls (*P* < 0.05)	↓ at 24 h	
				VEGF-C	P49767		Control	24 h			
							HIE	6 h, 24 h, 48 h, 72 h, 96 h	Decreased levels compared to healthy controls (*P* < 0.05)	↓ at 24 h	
				BDNF	P23560		Control	24 h			
							HIE		Decreased levels compared to healthy controls (*P* < 0.05)	↓ at 24 h	
				Fib-1C	P23142		Control	24 h			
							HIE	6 h, 24 h, 48 h, 72 h, 96 h	Decreased levels compared to healthy controls (*P* < 0.05)	↓ at 24 h	
				IGFBP-6	P24592		Control	24 h			
							HIE	6 h, 24 h, 48 h, 72 h, 96 h	Decreased levels compared to healthy controls (*P* < 0.05)	↓ at 24 h	
							HIE (with brain injury)	6 h, 24 h, 48 h, 72 h, 96 h	Decreased levels compared to HIE without brain injury (*P* < 0.05)	↓ at 72 h	
										↓ at 96 h	
				FasL	P48023		Control	24 h			
							HIE	6 h, 24 h, 48 h, 72 h, 96 h	Decreased levels compared to healthy controls (*P* < 0.05)	↓ at 24 h	
							HIE (with brain injury)		Decreased levels compared to HIE without brain injury (*P* < 0.05)	↓ at 72 h	
				FasR	P25445		Control	24 h			
							HIE	6 h, 24 h, 48 h, 72 h, 96 h	Increased levels compared to healthy controls (*P* < 0.05)	↑ at 24 h	
				Ang-2	O15123		HIE (with brain injury)	6 h, 24 h, 48 h, 72 h, 96 h	Decreased levels compared to HIE without brain injury (*P* < 0.05)	↓ at 24 h	
										↓ ↓ at 96 h	
				Hepsin	P05981		HIE (with brain injury)	6 h, 24 h, 48 h, 72 h, 96 h	Decreased levels compared to HIE without brain injury (*P* < 0.05)	↓ at 6 h	
										↓ at 24 h	
				HB-EGF	Q99075		HIE (with brain injury)	6 h, 24 h, 48 h, 72 h, 96 h	Decreased levels compared to HIE without brain injury (*P* < 0.05)	↓ at 24 h	
										↓ at 48 h	
				NP-1	O14786		HIE (with brain injury)	6 h, 24 h, 48 h, 72 h, 96 h	Decreased levels compared to HIE without brain injury (*P* < 0.05)	↓ at 24 h	
										↓ at 48 h	
										↓ at 96 h	
				ErbB3	P21860		HIE (with brain injury)	6 h, 24 h, 48 h, 72 h, 96 h	Decreased levels compared to HIE without brain injury (*P* < 0.05)	↓ at 24 h	
										↓ at 48 h	
				YKL-40	P36222		HIE (with brain injury)	6 h, 24 h, 48 h, 72 h, 96 h	Decreased levels compared to HIE without brain injury (*P* < 0.05)	↓↓ at 24 h	
										↓↓ at 48 h	
				IGFBP-1	P08833		HIE (with brain injury)	6 h, 24 h, 48 h, 72 h, 96 h	Decreased levels compared to HIE without brain injury (*P* < 0.05)	↓ at 24 h	
										↓↓ at 48 h	
				IGFBP-4	P22692		HIE (with brain injury)	6 h, 24 h, 48 h, 72 h, 96 h	Decreased levels compared to HIE without brain injury (*P* < 0.05)	↓ at 24 h	
										↓ at 48 h	
				IGFBP-5	P24593		HIE (with brain injury)	6 h, 24 h, 48 h, 72 h, 96 h	Decreased levels compared to HIE without brain injury (*P* < 0.05)	↓ at 6 h	
										↓ at 24 h	
										↓ at 48 h	
				Sortilin	Q99523		HIE (with brain injury)	6 h, 24 h, 48 h, 72 h, 96 h	Decreased levels compared to HIE without brain injury (*P* < 0.05)	↓ at 24 h	
										↓ at 48 h	
				MMP-3	P08254		HIE (with brain injury)	6 h, 24 h, 48 h, 72 h, 96 h	Decreased levels compared to HIE without brain injury (*P* < 0.05)	↓↓ at 72 h	
				Cathepsin-D	P07339		HIE (with brain injury)	6 h, 24 h, 48 h, 72 h, 96 h	Decreased levels compared to HIE without brain injury (*P* < 0.05)	↓ at 72 h	
				Endoglin	P17813		HIE (with brain injury)	6 h, 24 h, 48 h, 72 h, 96 h	Decreased levels compared to HIE without brain injury (*P* < 0.05)	↓ at 96 h	
				ICAM-1	P05362		HIE (with brain injury)	6 h, 24 h, 48 h, 72 h, 96 h	Decreased levels compared to HIE without brain injury (*P* < 0.05)	↓ at 96 h	
				MSP	P26927		HIE (with brain injury)	6 h, 24 h, 48 h, 72 h, 96 h	Decreased levels compared to HIE without brain injury (*P* < 0.05)	↓ at 6 h	
				TNFR-2	P20333		HIE (with brain injury)	6 h, 24 h, 48 h, 72 h, 96 h	Decreased levels compared to HIE without brain injury (*P* < 0.05)	↓ at 48 h	
										↓ at 72 h	
				NrCAM	Q92823		HIE (with brain injury)	6 h, 24 h, 48 h, 72 h, 96 h	Decreased levels compared to HIE without brain injury (*P* < 0.05)	↓ at 96 h	
				Fibronectin	P02751		HIE (with brain injury)	6 h, 24 h, 48 h, 72 h, 96 h	Decreased levels compared to HIE without brain injury (*P* < 0.05)	↓↓ at 48 h	
										↓↓ at 72 h	
				TIMP-1	P01033		HIE (with brain injury)	6 h, 24 h, 48 h, 72 h, 96 h	Decreased levels compared to HIE without brain injury (*P* < 0.05)	↓ at 48 h	
										↓ at 72 h	
										↓ at 96 h	
				TRAIL-R3	O14798		HIE (with brain injury)	6 h, 24 h, 48 h, 72 h, 96 h	Increased levels compared to HIE without brain injury (*P* < 0.05)	↑↑ at 6 h	
Metabolites											
Bersani, Ferrari et al. 2019		[44]	Plasma	Glucose	HMDB0304632	–	Moderate HIE (with hypothermia)	At birth			
							Severe HIE (with hypothermia)	At birth	No statistical differences (*P* > 0.05)	= on all groups	
Pineiro-Ramos, Nunez-Ramiro et al. 2020		[24]	Plasma	Lactate	HMDB0000190; HMDB0001311	GC–MS	HIE (normal MRI)	Pre-TH, 24 h, 48 h, 72 h			
							HIE (pathological MRI)	Pre-TH, 24 h, 48 h, 72 h	Increased levels compared to HIE with normal MRI (*P* < 0.05)	↑ at 24 h	
										↑ at 48 h	
				Pyruvate	HMDB0000243		HIE (normal MRI)	Pre-TH, 24 h, 48 h, 72 h			
							HIE (pathological MRI)	Pre-TH, 24 h, 48 h, 72 h	Increased levels compared to HIE with normal MRI (*P* < 0.05)	↑ at 72 h	
				Lactate/pyruvate ratio	-		HIE (normal MRI)	Pre-TH, 24 h, 48 h, 72 h			
							HIE (pathological MRI)	Pre-TH, 24 h, 48 h, 72 h	No statistical differences/no clear trend		
				Alanine, aspartate, and glutamate metabolism	HMDB0000812; HMDB0060350; HMDB0000191; HMDB0000168		HIE (pathological MRI)	Pre-TH, 24 h, 48 h, 72 h	Altered pathway associated with pathological MRI outcome	Altered at 72 h	
					HMDB0006483; HMDB0000052						
					HMDB0000161; HMDB0000208						
					HMDB0000641; HMDB0000148						
					HMDB0000112; HMDB0001552						
					HMDB0001301; HMDB0000254						
					HMDB0001254; HMDB0001128						
				Arginine and proline metabolism	HMDB0002104; HMDB0001369		HIE (pathological MRI)	Pre-TH, 24 h, 48 h, 72 h	Altered pathway associated with pathological MRI outcome	Altered at 48 h and 72 h	
					HMDB0000641; HMDB0000214						
					HMDB0000191; HMDB0000052						
					HMDB0000148; HMDB0001138						
					HMDB0006456; HMDB0006488						
					HMDB0003357; HMDB0000162						
					METPA0228; HMDB0003411						
					HMDB0006875; HMDB0000725						
					METPA0212; HMDB0000064						
					HMDB0012265; HMDB0000562						
					HMDB0004225; METPA0313						
					HMDB0003464; HMDB0000112						
					METPA0359; HMDB0033458						
					METPA0384; HMDB0001199						
					HMDB0001301; HMDB0001080						
					HMDB0000988; HMDB0001185						
					HMDB0002064; HMDB0003681						
					HMDB0060460; HMDB0002234						
					HMDB0000134; HMDB0006272						
					HMDB0003355; METPA0471						
					HMDB0000271; HMDB0000745						
					HMDB0003705						
				Caffeine metabolism	HMDB0000299; HMDB0000292		HIE (pathological MRI)	Pre-TH, 24 h, 48 h, 72 h	Altered pathway associated with pathological MRI outcome	Altered at pre-TH timepoint	
				D-glutamine and D-glutamate metabolism	HMDB0003423; HMDB0003339		HIE (pathological MRI)	Pre-TH, 24 h, 48 h, 72 h	Altered pathway associated with pathological MRI outcome	Altered at 48 and 72 h	
					METPA0146; HMDB0000148						
					HMDB0000641; HMDB0000805						
					HMDB0000208						
				Limonene and pinene degradation	HMDB0003667; METPA0496		HIE (pathological MRI)	Pre-TH, 24 h, 48 h, 72 h	Altered pathway associated with pathological MRI outcome	Altered at 72 h	
					HMDB0003647; HMDB0035089						
					HMDB0003450; HMDB0003634						
					HMDB0004321						
				Lysine biosynthesis	HMDB0001370; HMDB0000510		HIE (pathological MRI)	Pre-TH, 24 h, 48 h, 72 h	Altered pathway associated with pathological MRI outcome	Altered at 48 h and 72 h	
					HMDB0000191; HMDB0012250						
					HMDB0000719; HMDB0012249						
					HMDB0012289; METPA0439						
					HMDB0012267; METPA0493						
					HMDB0001370; HMDB0000279						
					HMDB0003518; HMDB0000208						
					HMDB0000225; HMDB0012247						
					HMDB0012266; HMDB0001263						
					HMDB0060320						
				Lysine degradation	HMDB0003405; HMDB0000182		HIE (pathological MRI)	Pre-TH, 24 h, 48 h, 72 h	Altered pathway associated with pathological MRI outcome	Altered at 72 h	
					HMDB0001084; HMDB0000279						
					HMDB0001345; HMDB0001325						
					HMDB0062259; HMDB0000070						
					HMDB0000206; HMDB0001263						
					HMDB0000510; HMDB0000225						
					HMDB0001339; HMDB0000661						
					HMDB0012233; HMDB0012176						
					HMDB0012175; HMDB0012114						
					HMDB0012115; METPA0449						
					METPA0112; METPA0456						
					HMDB0012151; HMDB0062496						
					HMDB0059600; HMDB0012130						
					HMDB0003355; HMDB0012131						
				Nitrogen metabolism	HMDB0000159; HMDB0000158		HIE (pathological MRI)	Pre-TH, 24 h, 48 h, 72 h	Altered pathway associated with pathological MRI outcome	Altered at 72 h	
					HMDB0000929; METPA0417						
					HMDB0000191; HMDB0000168						
					HMDB0000148; HMDB0000641						
					HMDB0001123; HMDB0000099						
					HMDB0000742; HMDB0000455						
					HMDB0000177; HMDB0000045						
				Phenylalanine metabolism	C03589; HMDB0000159; HMDB0006236; METPA0060		HIE (pathological MRI)	Pre-TH, 24 h, 48 h, 72 h	Altered pathway associated with pathological MRI outcome	Altered at 24 h, 48 h and 72 h	
					HMDB0134042; HMDB0000205						
					METPA0264; HMDB0010715						
					C00423; HMDB0033752						
					HMDB0013677; HMDB0000714						
					HMDB0002641; HMDB0002035						
					HMDB0001587; HMDB0006344						
					HMDB0000254; HMDB0000134						
					HMDB0012225; HMDB0000512						
					HMDB0000500						
					HMDB0000020; HMDB0001895						
					HMDB0000158						
				Seleno amino acid metabolism	HMDB0003011; HMDB0000161		HIE (pathological MRI)	Pre-TH, 24 h, 48 h, 72 h	Altered pathway associated with pathological MRI outcome	Altered at pre-TH timepoint	
				Steroid hormone biosynthesis	HMDB0000063; HMDB0000037		HIE (pathological MRI)	Pre-TH, 24 h, 48 h, 72 h	Altered pathway associated with pathological MRI outcome	Altered at pre-TH, 24 h, 48 h, 72 h	
					HMDB0000319; HMDB0000067						
					HMDB0000253; HMDB0000077						
					HMDB0003818; HMDB0000016						
					HMDB0001547; HMDB0001830						
					HMDB0003759;						
					HMDB0000374						
					HMDB0002802; HMDB0000234						
					HMDB0000053; HMDB0003769						
					HMDB0000490; HMDB0000899						
					HMDB0000031; HMDB0000145						
					HMDB0002961; HMDB0001425						
					HMDB0000920; HMDB0003069						
					HMDB0060437; METPA0446						
miRNA											
Ponnusamy, Kapellou et al. 2016		[31]	Plasma	EDTA-blood	RNU6B	GC10P013220	TaqMan miRNA assay	HIE	18 h–19 h	No comparisons between groups were performed	
					Let7b	GC22P046119					
					miR-21	GC17P059841					

*sLOX-1* soluble lectin-like oxidized low density lipoprotein receptor-1, *NSE* neuron-specific enolase, *S100B* S100-calcium-binding protein-B, *MMP* matrix metalloprotein, *IL* interleukin, *GFAP* glial fibrillary acidic protein, *TNF* tumor necrosis factor, *FABP4* fatty-acid-binding protein 4, *KLK-5* kallikrein 5, *VEGF* vascular endothelial growth factor, *BDNF* brain-derived neurotrophic factor, Fib-1C Fibulin-1C, *IGFBP* insulin-like growth factor binding protein, *FasL* Fas Ligand, *FasR* Fas receptor, *Ang* angiotensin, *HB-EGF* heparin-binding epidermal growth factor, *NP-1* alpha-defensin-1, *YKL-40* chitinase-3-like protein 1, *ICAM-1* intercellular cell adhesion molecule-1, *MSP* macrophage stimulating protein, *TNFR-2* TNF receptor 2, *NrCAM* neuron cell adhesion molecule, *TIMP-1* tissue inhibitor of metalloproteinase-1, *TRAIL-R3* tumor necrosis factor-related apoptosis-inducing ligand receptor 3, *EDTA* ethylene diamine tetraacetic acid, *SIMOA* single molecular array, *ELISA* enzyme linked immunosorbent assay, *Epo* erythropoietin, *GC–MS* gas chromatography–mass spectrometer, *HIE* hypoxic–ischemic encephalopathy, *MRI* magnetic resonance imaging, *miRNA* microRNA, *TH* Therapeutic hypothermia

**Table 3:** Summary of major findings related to biomarkers present in serum

References			Sample type	Biomarker	Code	Technique	Group	Collection time	Results	Summary	Observations
Proteins											
Ennen, Huisman et al. 2011		[17]	Serum	GFAP	P14136	Electrochemiluminescence, Sandwich Immunoassay	Control	< 6 h, 24 h, 48 h, 72 h, 96 h			
							HIE	< 6 h, 24 h, 48 h, 72 h, 96 h	Increased levels compared to controls at 6 h (*P* = 0.032), 24 h (*P* = 0.013), 48 h, 72 h (*P* = 0.013), 96 h (*P* = 0.003)	↑ at 6 h	
										↑ at 24 h	
										↑ at 72 h	
										↑ at 96 h	
							HIE (abnormal MRI)	< 6 h, 24 h, 48 h, 72 h, 96 h, 120 h, 144 h, 168 h	Increased levels compared to controls at 24 h (*P* = 0.02), 48 h (*P* = 0.007), 96 h (*P* < 0.001), 120 h (*P* < 0.001), 144 h (*P* = 0.007), 168 h (*P* < 0.05)	↑ at 24 h	
										↑ at 48 h	
										↑ at 96 h	
										↑ at 120 h	
										↑ at 144 h	
										↑ at 168 h	
Lopez-Suarez, Concheiro-Guisan et al. 2019		[21]	Serum	NSE	P09104	ELISA	HIE	< 6 h, 48 h, 72 h	High on 3 days of hypothermia	-	Positive correlation with carnitine C4
									Correlation with an unfavorable outcome (*P* = 0.029)		
Douglas-Escobar, Yang et al. 2010		[22]	Serum	pNF-H	P12036	ELISA	Control	< 6 h	Increased levels compared to control (*P* = 0.051)	↑ at 6 h	
							HIE	< 6 h, 7 h–12 h, 13 h–24 h, 25 h–48 h, 49 h–72 h			Tendency to higher levels on HIE with abnormal MRI, when compared to normal MRI,
											Higher levels on newborns with basal ganglia/hippocampus/thalamus injury
				UCHL1	P09936		Control	< 6 h	No significant differences	= at 6 h	
							HIE	< 6 h, 7 h–12 h, 13 h–24 h, 25 h–48 h, 49 h–72 h			
Massaro, Jeromin et al. 2013		[25]	Serum	UCHL1	P09936	ELISA	HIE with no/mild MRI injury	Initiation, 12 h, 24 h, 72 h			
							HIE with severe MRI injury or died	Initiation, 12 h, 24 h, 72 h	Increased levels compared to HIE without brain injury at initiation (*P* = 0.005) and 72 h (*P* = 0.039)	↑ at 6 h	
										↑ at 72 h	
				GFAP	P14136		HIE with no/mild MRI injury	Initiation, 12 h, 24 h, 72 h			
							HIE with severe MRI injury or died	Initiation, 12 h, 24 h, 72 h	Increased levels compared to HIE without brain injury at 24 h (*P* = 0.003) and 72 h (*P* = 0.002)	↑ at 24 h	
										↑ at 72 h	
Massaro, Chang et al. 2012		[28]	Serum	NSE	P09104	ELISA	HIE (good outcome)	Initiation, 12 h, 24 h, 72 h			Baseline values provide discrimination of death or severe MRI-brain injury, at 72 h, levels were good predictors of death or neurological deficit
							HIE (bad outcome)	Initiation, 12 h, 24 h, 72 h	Higher levels on adverse outcomes (death, severe MRI injury or neurological deficit)		
				S100B	P04271		HIE (good outcome)	Initiation, 12 h, 24 h, 72 h			Baseline values provide discrimination of death or severe MRI-brain injury
							HIE (bad outcome)	Initiation, 12 h, 24 h, 72 h	Higher levels on adverse outcomes (death, severe MRI injury or neurological deficit)		
Saito, Shibasaki et al. 2016		[34]	Serum	CRP	P02741	Chemical analysis	HIE	24 h, 48 h, 72 h, 96 h, 120 h, 144 h, 168 h	Increased levels at 48 h, peaking at 96 h		
				IL-6	P05231		HIE	24 h, 48 h, 72 h, 96 h, 120 h, 144 h, 168 h	Increased levels at 24 h, peaking at 48 h		
				PCT	P01258		HIE	24 h, 48 h, 72 h, 96 h, 120 h, 144 h, 168 h	Levels peaked at 48 h		
Jones, Heep et al. 2018		[34]	Serum	Troponin-T	P45379	-	non-HIE (normal EEG)	< 6 h			
							HIE (abnormal EEG)		Increased levels compared to non-HIE (*P* = 0.004)	↑ at < 6 h	
				CK	P12277		non-HIE (normal EEG)	< 6 h			
							HIE (abnormal EEG)		No significant differences (*P* = 0.157)		
				CRP	P02741		non-HIE (normal EEG)	< 6 h			
							HIE (abnormal EEG)		No significant differences (*P* = 0.846)		
				LDH	P07195		non-HIE (normal EEG)	< 6 h			
							HIE (abnormal EEG)		No significant differences (*P* = 0.225)		
				ALT	Q8TD30		non-HIE (normal EEG)	< 6 h			
							HIE (abnormal EEG)		Increased levels compared to non-HIE (*P* = 0.004)	↑ at < 6 h	
				AST	P17174		non-HIE (normal EEG)	< 6 h			
							HIE (abnormal EEG)		No significant differences (*P* = 0.291)		
				GGT	Q9UJ14		non-HIE (normal EEG)	< 6 h			
							HIE (abnormal EEG)		No significant differences (*P* = 0.480)		
				Alk Phos	P05186		non-HIE (normal EEG)	< 6 h			
							HIE (abnormal EEG)		No significant differences (*P* = 0.102)		
Alshweki, Perez-Munuzuri et al. 2017		[35]	Serum	S100B	P04271	ELISA	HIE (favorable outcome)	24 h, 48 h, 72 h			
							HIE (unfavorable outcome)	24 h, 48 h, 72 h	No significant differences	= on all timepoints	
							HIE (deceased)	24 h, 48 h, 72 h	Higher levels compared to alive at 24 h (*P* = 0.032)	↑ at 24 h	
				NSE	P09104		HIE (favorable outcome)	24 h, 48 h, 72 h			
							HIE (unfavorable outcome)	24 h, 48 h, 72 h	Higher levels compared to the favorable outcome at 48 h (*P* = 0.019)	↑ at < 48 h	Higher levels on infants with abnormal PET (*P* = 0.015)
							HIE (deceased)	24 h, 48 h, 72 h	Higher levels compared to alive at 24 h (*P* = 0.001) and 72 h (*P* = 0.006)	↑ at < 24 h	
										↑ at < 72 h	
Massaro, Chang et al. 2014		[36]	Serum	S100B	P04271	ELISA	Normal HIE (MDI and PSD > 85)	Initiation, 12 h, 24 h, 72 h			
							Mild/moderate HIE (MDI and PSD 70–85)	Initiation, 12 h, 24 h, 72 h			
							Severe HIE (MDI and PSD < 70)	Initiation, 12 h, 24 h, 72 h	Higher levels associated with worse cognitive (*P* = 0.07) and motor (*P* = 0.012) outcomes at 72 h		
							HIE (dead)	Initiation, 12 h, 24 h, 72 h		↑ at < 72 h	ROC curve predicts death or BSID-II MDI or PDI < 70
				NSE	P09104		Normal HIE (MDI and PSD > 85)	Initiation, 12 h, 24 h, 72 h			
							Mild/moderate HIE (MDI and PSD 70–85)	Initiation, 12 h, 24 h, 72 h			
							Severe HIE (MDI and PSD < 70)	Initiation, 12 h, 24 h, 72 h	Higher levels associated to worse cognitive (*P* = 0.010) and motor (*P* = 0.010) outcomes at 72 h		
							HIE (dead)	Initiation, 12 h, 24 h, 72 h		↑ at < 72 h	ROC curve predicts death or BSID-II MDI or PDI < 70
Fredly, Nygaard et al. 2016		[37]	Serum	CRP	P02741	-	HIE with low CRP levels	Initiation, 12 h, 24 h, 36 h, 48 h, 60 h, 72 h, 84 h, 96 h, 108 h, 120 h, 132 h, 144 h, 156 h, 168 h			
							HIE with high CRP levels		High levels in 82% of the infants studied		
Sweetman, Onwuneme et al. 2017		[38]	Serum	VEGF	P49767	Multiplex Cytokine Analysis	Controls	24 h, 48 h, 72 h, 96 h			
							HIE with no/mild injury	24 h, 48 h, 72 h, 96 h			
							HIE with moderate/severe injury	24 h, 48 h, 72 h, 96 h	Decreased levels compared to HIE with no/mild injury at 24 h (*P* = 0.035)	↓ at 24 h	Decreased levels at 24 h associated to death (*P* = 0.03)
				Erythropoietin	P01588		Controls	24 h, 48 h, 72 h, 96 h			
							HIE with no/mild injury	24 h, 48 h, 72 h, 96 h			
							HIE with moderate/severe injury	24 h, 48 h, 72 h, 96 h	Increased levels compared to HIE with no/mild injury at 48 h (*P* = 0.006)	↑ at 48 h	Increased levels at 72 h associated to death (*P* = 0.02)
Ezgu, Atalay et al. 2002		[39]	Serum	NSE	P09104	ELISA	No HIE	< 72 h	No significant differences	= at < 72 h	
							HIE with mild injury				
							HIE with moderate injury				
							HIE with severe injury				
El-Mazary, Abdel-Aziz et al. 2015		[40]	Serum	Hemoglobin	P69891	Chemical analysis	Control	< 48 h			
							HIE		Decreased levels compared to control (*P* = 0.001)	↓ at 48 h	
				ALT	Q8TD30		Control	< 48 h			
							HIE		Increased levels compared to control (*P* = 0.001)	↑ at 48 h	
				AST	P17174		Control	< 48 h			
							HIE		Increased levels compared to control (*P* = 0.001)	↑ at 48 h	
Metabolites											
El-Mazary, Abdel-Aziz et al. 2015		[40]	Serum	Urea	HMDB0000294	Chemical analysis	Control	< 48 h			
							HIE		Increased levels compared to control (*P* = 0.001)	↑ at < 48 h	
				Creatinine	HMDB0000562		Control	< 48 h			
							HIE		Increased levels compared to control (*P* = 0.001)	↑ at < 48 h	
Chouthai, Sobczak et al. 2015		[46]	Serum	Glucose	HMDB0304632	–	HIE (no TH)	Admission, < 24 h, 24 h–48 h, 48 h–72 h, 72 h–96 h			
							HIE (with TH)		Increased levels compared to HIE without TH (*P* = 0.025)	↑ at < 24 h	
							HIE with moderate/severe injury		Increased levels compared to HIE with mild or normal outcomes (*P* = 0.005)	↑ at < 24 h	High levels (> 200) associated with abnormal neuroimaging or death (*P* = 0.025)
Jones, Heep et al. 2018		[34]	Serum	Lactate	HMDB0000190; HMDB0001311	–	non-HIE (normal EEG)	< 6 h			
							HIE (abnormal EEG)		No significant differences (*P* = 0.07)	= at 6 h	
				Glucose	HMDB0304632		non-HIE (normal EEG)	< 6 h			First glucose measurement
							HIE (abnormal EEG)		Higher levels compared to non-HIE (*P* = 0.02)	↑ at 6 h	
Fredly, Nygaard et al. 2016		[37]	Serum	Lactate	HMDB0000190; HMDB0001311		HIE with low CRP levels	Initiation, 12 h, 24 h, 36 h, 48 h, 60 h, 72 h, 84 h, 96 h, 108 h, 120 h, 132 h, 144 h, 156 h, 168 h			
							HIE with high CRP levels		High levels compared to low-CRP group at 72 h (*P* = 0.004) and 96 h (*P* = 0.046)	↑ at 72 h	
										↑ at 96 h	
Electrolytes											
El-Mazary, Abdel-Aziz et al. 2015		[40]	Serum	Sodium	–	Chemical analysis	Control	< 48 h			
							HIE		Decreased levels compared to control (*P* = 0.001)	↓ at < 48 h	
				Potassium	–		Control	< 48 h			
							HIE		Increased levels compared to control (*P* = 0.001)	↑ at < 48 h	
				Calcium	–		Control	< 48 h			
							HIE		Decreased levels compared to control (*P* = 0.001)	↓ at < 48 h	
				Selenium	–	Atomic flame spectrophotometer	Control	< 48 h			
							HIE		Decreased levels compared to control (*P* = 0.001)	↓ at < 48 h	
							HIE with mild injury	< 48 h	No statistical differences (*P* = 0.05)		
							HIE with moderate injury	< 48 h	Decreased levels compared to control (*P* = 0.001)	↓ at < 48 h	
							HIE with severe injury	< 48 h	Decreased levels compared to control (*P* = 0.001)	↓ ↓ at < 48 h	

*GFAP* glial fibrillary acidic protein, *NSE* neuron-specific enolase, *pNF-H* phosphorylated neurofilament heavy chain, *UCHL1* ubiquitin C-terminal hydrolase L1, *S100B* S100-calcium-binding protein-B, *CRP* C-reactive protein, *PCT* procalcitonin, *IL* interleukin, *CK* creatine kinase, *LDH* lactate dehydrogenase, *ALT* alanine aminotransferase, *AST* aspartate aminotransferase, *GGT* γ-glutamyl transpeptidase, *VEGF* vascular endothelial growth factor, *ELISA* enzyme linked immunosorbent assay, *HIE* hypoxic–ischemic encephalopathy, *MRI* magnetic resonance imaging, *EEG* electroencephalogram, *PSD* particle size distribution, *MDI* metered dose inhaler, *ROC* receiver operating characteristic, *BSID-II* Bayley scales of infant development II

### Advanced cerebral imaging techniques are emerging as a therapeutic response monitoring approach

A small number of studies have evaluated the therapeutic response of newborns to hypothermia by evaluating the redox state of cytochrome oxidase [26, 41] or hemoglobin oxygenation [26, 30, 41] using near-infrared spectroscopy (NIRS) (Supplementary Table 3). In addition, the lactate/N-acetylaspartate ratio (assessed by ^1^H MRS) was suggested as a promising severity predictor for HIE [26, 41]. However, additional studies are needed for more robust conclusions.

### Several proteins as candidates for the diagnosis of hypoxic–ischemic encephalopathy are altered in various body fluids

**Fig. 2:**
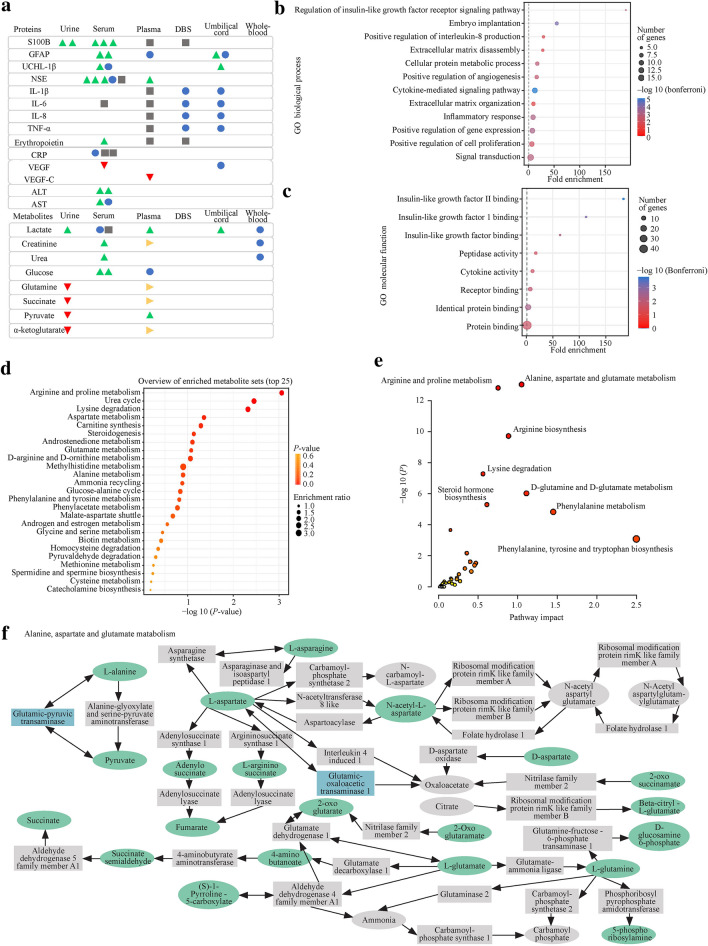
Proteins and metabolites identified as potential biomarkers. **a** Representation of biomarkers that were present in more than one fluid and/or more than one study. Green, red and yellow triangles represent studies with evidence of significantly increased, decreased, or altered levels of the molecule in the HIE group. Blue circles represent molecules without significant differences, and on gray squares, no comparison was performed. Plasma and serum samples were then combined for pathway analysis. **b**, **c** Gene ontology analysis of proteins, namely, biological process and molecular function. Altered pathways were identified using a metabolite enrichment analysis (**d**) and a combined approach of protein and metabolite analysis (mixomics approach) (**e**). **f** Alterations in the alanine, aspartate and glutamate pathways were identified, where metabolites are highlighted in green and proteins in blue. Balls represent metabolites, and squares represent proteins. *S100B* S100-calcium-binding protein-B, *DBS* dried blood spots, *GFAP* glial fibrillary acidic protein, *UCHL-1* ubiquitin C-terminal hydrolase L1, *NSE* neuron-specific enolase, *IL* interleukin, *CRP* C-reactive protein, *VEGF* vascular endothelial growth factor, *ALT* alanine aminotransferase, *AST* aspartate aminotransferase, *TNF-α* tumor necrosis factor α, *GO* Gene Ontology

The proteins identified in more than one body fluid and/or cited in more than one study are summarized in Fig. 2a. While serum appears to be the most studied sample type, in which eleven different proteins were identified as potential biomarkers, only one protein was identified in urine—S100-calcium-binding protein-B (S100B). This protein was reported to be elevated in newborns' urine, presenting an unfavorable outcome in the first 48 hours of life [35] and severe cases in the first 24 hours of life [44]. Accordingly, higher S100B serum levels during hypothermia (at 72 hours) were associated with a worse prognosis [28, 36] and even death [28, 35, 36]. For glial fibrillary acidic protein (GFAP), data available from the umbilical cord are not consensual: one study reported no differences between control and HIE [17], while another describes that elevated GFAP levels are significantly different in infants who developed severe brain injury compared to mild HIE [18]. Although no differences were found between control and HIE on plasma samples during TH and on rewarming [29], GFAP serum levels were described to be significantly higher in infants with abnormal MRI during the first 24 hours [17, 25]. In severe cases of HIE, ubiquitin C-terminal hydrolase L1 (UCHL-1) levels were described to be increased in umbilical cord plasma, but no differences were found when comparing moderate and severe HIE groups with the mild HIE group [18]. It is also unclear if serum levels of this protein are associated with a worse prognosis, since no differences were found between the HIE and control groups [22], but the levels were described to be elevated at 6 hours and 72 hours in newborns with severe MRI brain injury when compared with those who did not develop or developed a mild brain injury [25]. Interestingly, newborns with HIE showed higher levels of plasma neuron-specific enolase (NSE) before TH than controls [29]. Increased serum levels of NSE in the first 72 hours were also found to be positively correlated with an unfavorable outcome [28, 35, 36], whereas one study did not find differences between newborns with different severity grades [39], and another did not have a control group [21].

Concerning the identified cytokines, no significant differences were found either in the umbilical cord serum [18] or the DBS [32]. Particularly for IL-6 serum levels, no control group was available in the study [33]. No comparisons were performed on erythropoietin levels in plasma or DBS [32]. However, a study reported that erythropoietin was increased in serum at 48 hours in newborns with moderate/severe HIE when compared to those who did not develop or developed mild HIE [38]. In addition, this study associated increased levels of erythropoietin at 72 hours with death [38]. C-reactive protein (CRP) serum levels and their correlation to an outcome are unclear, since one study did not find significant differences [34], and other studies did not have control groups to compare [33], or their levels were associated with microcirculatory issues [37]. Vascular endothelial growth factor (VEGF) serum levels were decreased in newborns with moderate/severe injury at 24 hours [38], a tendency also verified in plasma samples, where VEGF-C was decreased at 24 hours compared to controls [42]. Levels of VEGF in umbilical cord serum did not vary significantly [18].

Finally, alanine aminotransferase (ALT) serum levels were increased in HIE newborns with an abnormal electroencephalogram (EEG) in the first 6 hours of life [34] and continued to increase at 48 hours compared to the control group [40]. Aspartate aminotransferase (AST) levels were also reported to be elevated in newborns with HIE at 48 hours [40], but no difference was found in the first 6 hours of life [34]. However, it should be taken into consideration that they lack neurological specificity [47].

Together, these data suggest that additional studies are still required to corroborate the findings presented in this review regarding GFAP, UCHL-1, ALT, and VEGF. Concerning urinary S100B, receiver operating characteristic (ROC) curves show high sensitivity and specificity to predict death and short outcomes [35]. In addition, S100B and NSE serum levels also showed good predictive power for short- [28] and long-term outcomes [36]. Therefore, these two proteins should be validated to provide further support in the diagnosis of HIE.

Metabolites are promising candidates for the diagnosis of hypoxic–ischemic encephalopathy but require further studies*. *The metabolites identified and altered in more than one body fluid are summarized in Fig. 2a. Plasma is the sample type that reported more biomarkers, while umbilical cord blood metabolites were only studied by one research group. The most studied metabolite was lactate, which was reported to be increased in urine in the first 6 hours of life on HIE compared to controls [43]. In addition, HIE newborns with a severe phenotype or pathological MRI showed increased lactate levels in the cord blood [45] and plasma [24] at birth, respectively. However, the levels of this metabolite were reported not to change significantly in whole blood [27] or serum [34] in the first hours of life. Creatinine and urea are reported to be elevated in serum samples of the HIE group at 48 hours [40] but not in whole blood at birth [44]. In plasma, creatinine was reported to be significantly altered [24].

Interestingly, glucose levels were reported to be significantly increased in the serum of newborns in the first hours of life with severe injury or abnormal EEG [34, 46], but Bersani and his team did not find differences in plasma levels [44]. Last, glutamine, succinate, pyruvate and α-ketoglutarate urine levels were found to be decreased in HIE patients in the first 6 hours of life [43], whereas in plasma, glutamine and α-ketoglutarate were reported to be altered at 48 hours and 72 hours, and succinate was altered at 24 hours, 48 hours, and 72 hours [24]. Regarding pyruvate, its levels were elevated at 72 hours in newborns who presented a pathological MRI [24]. Furthermore, there are studies that propose ratios of metabolites as putative biomarkers. In urine, the lactate/creatinine ratio was described to be significantly elevated in the first 24 hours of life [23], but no differences were found in the lactate/pyruvate ratio [24] in plasma or the free/total carnitine ratio in DBS [21]. Inconsistencies and the reduced number of studies impaired the identification of any metabolite as a putative biomarker. Nevertheless, lactate is a candidate that should be explored in future studies along with new in-depth screenings, considering that it is a systemic severity marker but not neuronal-specific [48].

### New approaches to identify other classes of biomarkers

MicroRNAs (miRNAs) were studied as potential biomarkers [31]. However, no differences were found between HIE patients with favorable and unfavorable outcomes in Let7b, miR-21, miR-29b, miR-124, and mir-155 levels in DBS. Alterations in the number of blood cells were also evaluated as an approach to identify biomarkers. Nucleated red blood cells in the cord blood were increased in newborns with moderate/severe HIE [45], but no differences were found in neutrophils or white blood cells in the whole blood [33, 40]. Platelet levels did not show significant differences when comparing newborns with high CRP levels [33] but were decreased in the HIE group compared to the control group [40]. Furthermore, electrolyte serum levels were also studied. One study showed that sodium, calcium, and selenium levels were decreased in the HIE group, while potassium levels were increased [40]. Finally, studies concerning alterations in the abundance of RNA for several inflammation markers were also reported. Proliferator-activated receptor gamma (PPARG), matrix metallopeptidase 9 (MMP-9), interleukin (IL)-8, heat shock protein family A (Hsp70) member 1A (HSPA1A), and toll-like receptor 8 (TLR8) were found to be increased in the whole blood of the HIE group, while C–C motif chemokine receptor 5 (CCR5) was decreased [20]. Since these approaches to identify biomarkers are recent, due to the lack of corroborating evidence from different authors, no objective conclusion can be drawn about the use of the aforementioned analytes as potential biomarkers.

### Plasma and serum potential biomarkers showed altered pathways in hypoxic–ischemic encephalopathy

Pieces of evidence from plasma and serum were combined for further pathway analysis to highlight potential mechanisms. Gene Ontology (GO) of extracted proteins showed several biological processes and molecular functions associated with inflammatory responses, as well as insulin-growth factor pathways (Fig. 2b, c). Specifically, the majority of the proteins associated with these ontologies were decreased in HIE newborns [42]. Concerning metabolite pathways, arginine and proline metabolism, urea cycle and lysine degradation were the most significantly altered (Fig. 2d). Finally, integrating protein and metabolite data, two pathways were identified as being modified in HIE: alanine, aspartate and glutamate metabolism and arginine and proline metabolism (Fig. 2e, f).

## Discussion

Presently, no accurate tools are available to diagnose HIE immediately after birth and quickly define the best therapeutic approach. In addition, the guidelines for diagnosing HIE are not standardized among all pediatric centers. While verifying the eligibility criteria of each study, we found huge discrepancies between the parameters applied to diagnose HIE, assessment of severity, and time of sample collection. Together, this reinforces the need to establish standard diagnostic criteria worldwide. In this review, to reduce bias, the population was homogenized by (1) matching at least two ACOG diagnosis criteria and (2) having neuroimaging evidence of brain injury, since these data are unequivocal proof of brain damage. In addition, potentially biased studies were not excluded, since they did not focus on the major findings of this review.

This systematic review summarized the potential biomarkers for HIE. Reported results lack high-throughput screenings, which hampers the identification of a larger number of putative biomarkers. Briefly, serum is the most cited fluid, and proteins are the major candidates with more consistent results among the different studies. In particular, NSE and S100B were identified as potential biomarkers for HIE. Interestingly, not only these proteins but also UCHL-1 and GFAP were described as potential biomarkers for traumatic brain injury in adults [49], since they are also involved in brain damage mechanisms. NSE is a brain and peripheral neuroendocrine-specific enolase that is highly expressed in neurons [50]. The postinsult collapse of the plasma membrane, as after a perinatal asphyxia event, could cause the release of this protein to peripheral fluids. In particular, after ischemic stroke, NSE protein levels were positively correlated with the extent of brain damage [51]. However, it should be considered that altered levels of this protein might also be associated with the diagnosis of small cell lung cancer, among others [52]. S100B is a calcium-binding protein expressed by glia, especially astrocytes [53]. This protein is associated with intracellular structures, but it is also secreted, playing a role in cell survival (in nanomolar concentrations), apoptosis, lipid peroxidation (in micromolar concentrations) and cytokine production [53, 54]. In addition, a highlighted pathway in GO analysis was the insulin-like growth factor signaling pathway (Fig. 2b, c). Although a single research group [42] studied the identified proteins, this is a promising target, since Insulin-like growth factor 1 (IGF-1) has already been tested as a therapeutic approach, exhibiting good outcomes in a rat HIE model [55].

Regarding the direct inflammatory response, no differences were found in the analysis of a panel of cytokines at the protein level [18, 32]. However, when analyzing the RNA levels of inflammatory markers, five were increased, and one was decreased [33]. GO analysis also identified alterations in several inflammatory pathways (Fig. 2b, c). However, some proteins have opposite tendencies, reinforcing the need to clarify the relevance of these molecules in the diagnosis of HIE. Nevertheless, it should be taken into consideration that the elevation of IL-6, for example, has been described to be associated with neonatal sepsis [56], which could lead to a misleading diagnosis.

The alanine, aspartate, and glutamate pathways were found to be altered in newborns with HIE. These metabolites were already described to be increased in brain tissue in a rat model of traumatic brain injury [57]. However, it should be taken into consideration that dysregulation of alanine transaminase was associated with liver dysfunction in HIE [58], enhancing the need to consider systemic biomarkers. Glutamate and alanine were also described to be elevated in the CSF in an HIE piglet model [58], while an excitotoxicity mouse model identified proline and arginine as players in response to injury [59]. Based on these pieces of evidence, future studies should focus on the characterization of these pathways in HIE.

Studies using advanced imaging techniques, such as NIRS and ^1^H MRS, are emerging as promising noninvasive approaches to monitor newborns’ response to TH [26, 27, 30, 37, 41]. However, they refer to a low number of publications, focusing on a low number of patients and without healthy controls, and were classified as potentially biased according to the NOS scale. Although these techniques can provide more information about the metabolic state of the newborns, access to these specialized techniques might be easier at reference centers to treat HIE but not in all maternities due to financial and logistical reasons.

One of the weak points of this review is the lack of studies with healthy newborn controls (Fig. 1i). Due to ethical reasons, it is not possible to obtain samples from healthy newborns at several timepoints. As an alternative, studies use non-neurological brain-injured newborns or newborns who have suffered an episode of perinatal asphyxia but did not develop/or developed a mild brain injury. In either case, using these populations as controls can bias the conclusions. Another limitation is the lack of uniformization of the groups between the studies (Fig. 1i), which makes it more difficult to compare studies. Likewise, the lack of uniformity of sample collection time, which might be influenced by hypothermia, hindered drawing conclusions. Unfortunately, some of the studies analyzed lack transparency on the methodologies and the availability of raw data, which compromised data extraction and further analysis of the published data.

It should be noted that only a small number of studies performed screenings, which limits the amount of information extracted from the samples and, therefore, reduces the chances of identifying a biomarker. In addition, the lack of raw data available (even after direct request) impaired a more detailed analysis to determine the sensitivity and specificity of the identified biomarkers and assess their predictive value to diagnose HIE and/or predict its severity. Therefore, future studies should present a higher consistency in the diagnosis criteria, establishment of groups, preferably using healthy controls, and sample collection time, so that data presented in this manuscript can be corroborated and finally get to a routine clinical application.

In conclusion, elevated serum levels of NSE and S100B correlated with a worse prognosis in newborns suffering from HIE. Nevertheless, future studies should focus on determining the sensitivity and specificity of these molecules before entering clinical practice. In addition, since other molecules were identified as potential biomarkers, such as GFAP, UCHL1, ALT, glutamate and lactate, we suggest that future studies focus on identifying a panel of biomarkers instead of a standalone biomarker.

## Supplementary Information

Below is the link to the electronic supplementary material.

Supplementary file1 (PDF 90 KB)

Supplementary file2 (XLSX 311 KB)

Supplementary file3 (XLSX 216 KB)

Supplementary file4 (XLSX 127 KB)

Supplementary file5 (DOCX 81 KB)

## Data Availability

All the data is provided on the tables of the manuscript, as well as in the supplementary data.
