# Supplementary material for: Biomarkers of hypoxic–ischemic encephalopathy: a systematic review
Source: World J Pediatr. 2023 Apr 21;19(6):505–48. doi: 10.1007/s12519-023-00698-7 (PMC10199106; doi:10.1007/s12519-023-00698-7)
Supplement: Supplementary file 1 — Supplementary file1 (PDF 90 KB) [file 12519_2023_698_MOESM1_ESM.pdf]

Methods in detail

Search strategy

Since HIE terminology is not consensual, four different terms for the disease were searched. All terms were combined with the preposition “and”. In PubMed, the search included the terms “neonatal brain injury”, “neonatal encephalopathy”, “hypoxic-ischemic encephalopathy” and “neonatal hypoxic-ischemic encephalopathy” in the Title/Abstract field, combined with the terms “biomarker*”, “proteomic*”, “metabolomic*” on the MeSH Terms field. In Web of Science, the searched terms were “neonatal brain injury”, “neonatal encephalopathy”, “hypoxic-ischemic encephalopathy” and “neonatal hypoxic-ischemic encephalopathy” on the Title field, combined with the terms “biomarker*”, “proteomic*”, “metabolomic*” on the Topic field. Here, results were restricted by language (English) and document type (Article). Finally, in the Science Direct database, the search comprised the terms “neonatal brain injury”, “neonatal encephalopathy”, “hypoxic-ischemic encephalopathy” and “neonatal hypoxic-ischemic encephalopathy” on the Title field combined with the terms “biomarker”, “proteomics”, “metabolomics” on the Title, Abstract or author-specified keywords field. For grey literature, the OpenGrey database was used and the four different terminologies previously used were combined with “biomarker*”, “proteomic*” and “metabolomic*”. Articles suggested by the authors resulting from the manual search were also accepted.

Duplicates resulting from different search words and from distinct databases were excluded.

Study eligibility

Selected articles were subjected to abstract evaluation and three selection phases. The first approach aimed to categorize the results by document type (book chapter, case report,

commentary, conference paper/abstract, editorial, letter, oral presentation, original paper, poster, protocol or undefined), language (English and non-English), species of the samples studied (cell line, foal, human, lamb, piglet, primate, rodent, sheep or undefined) and biomarker type (e.g., biochemical, genetic, biochemical associated to other diseases than HIE, pH, non-biochemical, therapeutic response or undefined). Only English original papers in which the research was focused on biochemical HIE biomarkers in human samples were selected for methods evaluation. Articles were then classified according to study type (prospective, retrospective, case-control, randomized clinical trial or other), sample size, sample type (cord blood, CSF, imaging, serum, plasma, tissue, urine, whole-blood, or more than one), association to other pathologies, e.g., genetic or metabolic comorbidities (yes, no or undefined), gestational age (GA) (≥36 weeks GA, <36 weeks GA) or age (child > 1-year-old and adult > 18 years), disease (brain injury by other cases, HIE, perinatal asphyxia (PA), PA associated to HIE or other), sample collection time (within 72h before or during therapy, within 72h without therapy, within one week, within one month, post-mortem, multiple timepoints or other), therapy (whole-body hypothermia, head cooling, other or no information), outcomes assessment (within 6 months, 9 months, 12 months or no information) and association of the biomarkers to multiorgan failure (yes, no, or no information). Only studies regarding term newborns collected in the first 72h of life were selected for a diagnostic criteria analysis. Moreover, studies analyzing CSF were excluded since its collection from newborns may be considered unethical in many countries.

Since diagnosis criteria for HIE are not standardized in all studies, to analyze a homogenous population, the criteria applied were assessed in each study: APGAR score (threshold and time), fetal acidemia (pH and base deficit/base excess levels), MRI (if it was performed or not, or other complementary diagnostic exams) and multiple organ

failure (yes, no or no information). These parameters were then evaluated by ACOG diagnosis criteria, as previously described. Studies that matched at least two ACOG diagnosis criteria or had neuroimaging evidence of brain injury were selected for quality assessment and data extraction.

Data extraction

Population characteristics were analyzed to infer the homogeneity of the populations being reviewed in this manuscript. Information about the study location, type of study, gestational age and/or birth weight, diagnosis criteria, HIE severity assessment, complementary diagnostic exams, therapeutic hypothermia (and other pharmacological approaches), sample size, sample type, and the biomarker described in the study were extracted. In addition, information about the biomarker, namely the biomarker type (protein, metabolite, ion, RNA, miRNA, number of cells, or imaging), the technique used to analyze the biomarker, the sample size of each group, collection time, and p-value (when available) was extracted.
