# Supplementary material for: Biomarkers of hypoxic–ischemic encephalopathy: a systematic review
Source: World J Pediatr. 2023 Apr 21;19(6):505–48. doi: 10.1007/s12519-023-00698-7 (PMC10199106; doi:10.1007/s12519-023-00698-7)
Supplement: Supplementary file 5 — Supplementary file5 (DOCX 81 KB) [file 12519_2023_698_MOESM5_ESM.pdf]

Table 1 – Risk of bias assessment of cohort studies using the NOS scale.

COHORT STUDIES												
	Selection				Comparability	Outcome						
Reference	Representativeness of the exposed cohort	Selection of the non-exposed cohort	Ascertainment of exposure	Demonstration that outcome of interest was not present at start of study	Comparability of cohorts on the basis of the design or analysis	Assessment of outcome	Was follow-up long enough for outcomes to occur	Adequacy of follow up of cohorts	Selection	Comparability	Outcome	TOTAL
(Alshweki, Perez-Munuzuri et al. 2017)[35]	*	*	*	*		*	*	*	4	0	3	7
(Balada, Tebe et al. 2020)[20]	*		*	*	**	*	*	*	3	2	3	8
(Bale, Mitra et al. 2014)[41]	*		*	*		*	*	*	3	0	3	6
(Chalak, Sánchez et al. 2014)[18]	*	*	*	*	**	*	*	*	4	2	3	9
(Dehaes, Aggarwal et al. 2014)[30]	*		*	*	**	*	*	*	3	2	3	8
(Douglas-Escobar, Yang et al. 2010)[22]	*	*	*	*	*	*	*	*	4	1	3	8
(El-Mazary, Abdel-Aziz et al. 2015)[40]	*	*	*	*	**				4	2	0	6
(Ennen, Huisman et al. 2011)[17]	*		*		*	*	*	*	2	1	3	6
(Ezgu, Atalay et al. 2002)[39]	*	*	*		*	*	*	*	3	1	3	7
(Fredly, Nygaard et al. 2016)[37]	*	*	*		**	*	*	*	3	2	3	8
(Haiju, Suyuan et al. 2008)[45]	*	*	*	*	**	*		*	4	2	2	8
(Jain, Pagano et al. 2017)[27]	*	*	*	*	**	*	*	*	4	2	3	9
(Jones, Heep et al. 2018)[34]	*	*	*		**	*		*	3	2	2	7
(Locci, Noto et al. 2018)[43]	*	*	*	*	**	*	*	*	4	2	3	9
(Lopez-Suarez, Concheiro-Guisan et al. 2019)[21]	*	*	*	*	**	*	*	*	4	2	3	9
(Maggiotto, Sondhi et al. 2019)[29]	*	*	*	*	**	*	*	*	4	2	3	9
(Massaro, Chang et al. 2014)[36]	*	*	*	*	**	*	*		4	2	2	8

(Massaro, Chang et al. 2012)[28]	*	*	*	*	**	*	*	*	4	2	3	9
(Massaro, Jeromin et al. 2013)[25]	*	*	*	*	**	*	*	*	4	2	3	9
(Massaro, Wu et al. 2019)[32]	*	*	*	*		*	*	*	4	0	3	7
(Mitra, Bale et al. 2016)[26]		*				*	*		1	0	2	3
(Oh, Perritt et al. 2008)[23]	*	*	*	*	*	*	*	*	4	1	3	8
(Pineiro-Ramos, Nunez-Ramiro et al. 2020)[24]	*	*	*	*	**	*	*	*	4	2	3	9
(Ponnusamy, Kapellou et al. 2016)[31]	*	*	*	*		*	*	*	4	0	3	7
(Saito, Shibasaki et al. 2016)[33]	*	*	*	*		*			4	0	1	5
(Akamatsu, Sugiyama et al. 2019)[19]	*	*	*	*	*	*	*	*	4	1	3	8
(Shaikh, Boudes et al. 2015)[42]	*	*	*		**	*	*	*	3	2	3	8
(Sweetman, Onwuneme et al. 2017)[38]	*	*	*	*	**	*	*	*	4	2	3	9

Note: the scores represent the sum of the number of stars.

Table 2 – Risk of bias assessment of case-control studies using the NOS scale.

CASE-CONTROL STUDIES												
	**Selection**				**Comparability**	**Outcome**						
**Reference**	**Is the case definition adequate?**	**Representativeness of the cases**	**Selection of Controls**	**Definition of Controls**	**Comparability of cases and controls on the basis of the design or analysis**	**Ascertainment of exposure**	**Same method of ascertainment for cases and controls**	**Non-Response rate**	**Selection**	**Comparability**	**Outcome**	**TOTAL**
(Bersani, Ferrari et al. 2019)[44]	*			*	**	*	*	*	2	2	3	7
(Chouthai, Sobczak et al. 2015)[46]		*	*	*	**	*		*	3	2	2	7

Note: the scores represent the sum of the number of stars.

Table 3 – Summary of findings of biomarkers using imaging techniques.

Reference	Sample type	Name	Technique	Group	Collection time	Results	Summary	Observations
(Mitra, Bale et al. 2016) [26]	Cerebral imaging	Lactate/N-acetylasperatate<br>ΔoxCCO<br>(redox of cytochrome oxidase)<br>ΔHbD<br>(hemoglobin difference)	^{1}H MRS<br>NIRS	HIE	During TH			
(Jain, Pagano et al. 2017) [27]	Cerebral imaging	$CrSO_2$	NIRS	HIE	During TH; <48h	Higher absolute values between 24h and 36h associated to MRI abnormalities ($p=0.011$)<br>At 30h, increased levels correlated with basal ganglia ($p=0.001$), white-mater ($p=0.012$), PLIC ($p=0.001$) and brain stem ($p=0.03$)	↑ at 24h-36h	
(Dehaes, Aggarwal et al. 2014) [30]	Cerebral imaging	CBV (cerebral blood volume)	FDNIRS–DCS	Control<br>HIE (during TH)<br>HIE (pos TH)	During TH (<72h); Post-TH (<300h)	Increased levels compared to control ($p<10^{-3}$) and pos-TH ($p<0.01$)	↑ at <72h	
		CBF (cerebral blood flow)		Control<br>HIE (during TH)<br>HIE (pos TH)		Decreased levels compared to control ($p<0.01$) and pos-TH ($p<0.01$)	↓ at <72h	
		SO_2 (hemoglobin oxygen saturation)		Control<br>HIE (during TH)<br>HIE (pos TH)		Increased levels compared to control ($p=0.014$)	↑ at <72h	
		$CMRO_2$ (cerebral oxygen metabolism index)		Control<br>HIE (during TH)<br>HIE (pos TH)		Decreased levels compared to control ($p<10^{-5}$) and pos-TH ($p<0.01$)	↓ at <72h	
		HbT (total hemoglobin)		Control<br>HIE (during TH)		Increased levels compared to control ($p=0.01$)	↑ at <72h	
		HbO (oxyhemoglobin)		Control<br>HIE (during TH)		Increased levels compared to control ($p<0.01$)	↑ at <72h	
(Bale, Mitra et al. 2014) [41]	Cerebral imaging	Thalamic Lactate/N-acetylasparatate	^{1}H MRS	HIE	During <144h			Used as a biomarker of severity
		ΔSPO_2 (systemic oxygen saturation)	NIRS	HIE				
		ΔHbD<br>(oxygenated - deoxygenated hemoglobin)		HIE		Associated to Lac/NAA ratio ($p<0.01$)		
		ΔHbT (oxygenated + deoxygenated hemoglobin)		HIE				
		ΔoxCCO (redox of cytochrome oxidase)		HIE		Associated to Lac/NAA ratio ($p<0.01$)		
(Fredly, Nygaard et al. 2016) [37]	Cerebral imaging	erebral oxygenation	NIRS	HIE with low CRP levels<br>HIE with high CRP levels	24h, 48h, 72h<br>24h, 48h, 72h	No statistical differences		

Table 4 – Summary of findings of biomarkers present in urine.

Reference	Sample type	Name	Code	Technique	Group	Collection time	Results	Summary	Observations
**Proteins**									
(Alshweki, Perez-Munuzuri et al. 2017) [35]	Urine	S100B	P04271	ELISA	HIE (favorable outcome)	24h, 48h, 72h			
					HIE (unfavorable outcome)		Higher levels at 24h (p=0.031) and 48h (p=0.002)	↑ at 24h<br>↑ at 48h	Higher levels at 24h, 48h and 72h on infants who died;<br>Higher levels at 24h, 48h on infants with abnormal PET
(Bersani, Ferrari et al. 2019) [44]	Urine	S100B	P04271	ILMA	Moderate HIE (with hypothermia)	First void, 4h, 8h, 12h, 16h, 20h, 24h, 36h, 48h, 72h, 96h, 108h, 120h	Peak level at 4h (p<0.05)		Data is also available for groups that were not treated with TH
					Severe HIE (with hypothermia)		Increasing levels from first void, peaking at 16h (p<0.05)<br>Higher levels from 4h to 24h (p<0.001)	↑ at 4h<br>↑ at 8h<br>↑ at 12h<br>↑ at 16h<br>↑ at 20h<br>↑ at 24h	
**Metabolites**									
(Oh, Perritt et al. 2008) [23]	Urine	Lactate/ creatinine ratio	-	-	Infants with normal/mild disability<br>Infants with moderate/severe disability or death	6h-24h, 48h-72h	Higher levels at 6h-24h (p<0.001)	↑ at 6h-24h	No statistical differences between control and hypothermia groups
(Locci, Noto et al. 2018) [43]	Urine	Lactate	HMDB0 000190; HMDB0 001311	^{1}H NMR	Control	<6h			
					HIE	<6h, 48h, 72h, day 30	Increased levels compared to healthy controls at <6h	↑ at <6h	Higher lactate levels on non- surviving infants
		Myo-inositol	HMDB0 000211		Control	<6h			
					HIE	<6h, 48h, 72h, day 30	Increased levels compared to healthy controls at <6h	↑ at <6h	
		Betaine	HMDB0 000043		Control	<6h			
					HIE	<6h, 48h, 72h, day 30	Increased levels compared to healthy controls at <6h	↑ at <6h	
		Taurine	HMDB0 000251		Control	<6h			
					HIE	<6h, 48h, 72h, day 30	Increased levels compared to healthy controls at <6h	↑ at <6h	
		Citrate	HMDB0 000094		Control	<6h			
					HIE	<6h, 48h, 72h, day 30	Decreased levels compared to healthy controls at <6h	↓ at <6h	
		Acetone	HMDB0 001659		Control	<6h			
					HIE	<6h, 48h, 72h, day 30	Decreased levels compared to healthy controls at <6h	↓ at <6h	
		DMA	HMDB0 246062		Control	<6h			
					HIE	<6h, 48h, 72h, day 30	Decreased levels compared to healthy controls at <6h	↓ at <6h	
					Control	<6h			

		Glutamine	HMDB0003423; HMDB0000641		HIE	<6h, 48h, 72h, day 30	Decreased levels compared to healthy controls at <6h	↓ at <6h
		Succinate	HMDB0000254		Control	<6h		
					HIE	<6h, 48h, 72h, day 30	Decreased levels compared to healthy controls at <6h	↓ at <6h
		Pyruvate	HMDB0000243		Control	<6h		
					HIE	<6h, 48h, 72h, day 30	Decreased levels compared to healthy controls at <6h	↓ at <6h
		α-Ketoglutarate	HMDB0000208		Control	<6h		
					HIE	<6h, 48h, 72h, day 30	Decreased levels compared to healthy controls at <6h	↓ at <6h
		N-Acetyl groups	-		Control	<6h		
					HIE	<6h, 48h, 72h, day 30	Decreased levels compared to healthy controls at <6h	↓ at <6h
		Acetate	HMDB0000042		Control	<6h		
					HIE	<6h, 48h, 72h, day 30	Decreased levels compared to healthy controls at <6h	↓ at <6h
		Arginine	HMDB0000517; HMDB0003416		Control	<6h		
					HIE	<6h, 48h, 72h, day 30	Decreased levels compared to healthy controls at <6h	↓ at <6h
**miRNA**								
(Ponnusamy, Kapellou et al. 2016) [31]	Urine	RNU6B	GC10P013220	TaqMan miRNA assay	HIE	18h-19h	No comparisons between groups were performed	
		Let7b	GC22P046119					
		miR-21	GC17P059841					

Table 5 – Summary of findings of biomarkers present in umbilical cord blood.

Reference	Sample type	Name	Code	Technique	Group	Collection time	Results	Summary	Observations
**Proteins**									
(Ennen, Huisman et al. 2011) [17]	Cord blood	GFAP	P14136	Electrochemiluminescence Sandwich Immunoassay	Control	At birth			
					HIE		No significant differences	= at 0h	
(Chalak, Sánchez et al. 2014) [18]	Umbilical cord plasma	GFAP	P14136	ELISA	Mild HIE	At birth			
					Moderate HIE		Increased levels compared to mild HIE (p=0.001)	↑ at 0h	
					Severe HIE			↑↑ at 0h	
		UCHL-1	P09936		Mild HIE	At birth			
					Moderate HIE		Increased levels compared to mild HIE (p=0.03)	↑ at 0h	
					Severe HIE			↑↑ at 0h	
	Umbilical cord serum	GFAP	P14136	ELISA	Mild HIE	At birth			
					Moderate/severe HIE		Increased levels compared to mild HIE (p<0.05)	↑ at 0h	
		UCHL-1	P09936		Mild HIE	At birth			
					Moderate/severe HIE		No significant differences	= at 0h	
		IL-1	P01584		Mild HIE	At birth			
					Moderate/severe HIE		No significant differences	= at 0h	
		IL-6	P05231		Mild HIE	At birth			
					Moderate/severe HIE		No significant differences	= at 0h	
		IL-8	P10145		Mild HIE	At birth			
					Moderate/severe HIE		No significant differences	= at 0h	
		VEGF	P49767		Mild HIE	At birth			
					Moderate/severe HIE		No significant differences	= at 0h	
		IFN-Ɣ	P01579		Mild HIE	At birth			
					Moderate/severe HIE		No significant differences	= at 0h	
		TNF	P01375		Mild HIE	At birth			
					Moderate/severe HIE		No significant differences	= at 0h	
		RANTES	P13501		Mild HIE	At birth			
					Moderate/severe HIE		No significant differences	= at 0h	
**Metabolite**									
(Haiju, Suyuan et al. 2008) [45]	Cord blood	Lactate	HMDB0000190	Blood gas and biochemical analysis	Mild HIE	At birth			
					Moderate/severe HIE		Increased levels compared to mild HIE (p<0.05)	↑ at 0h	
**Number of cells**									
(El-Mazary, Abdel-Aziz et al. 2015) [40]	Serum	Platelet	-	Chemical analysis	Control	<48h			
					HIE		Decreased levels compared to control (p=0.01)	↓ at <48h	
		White blood cells			Control	<48h			
					HIE		No statistical differences (p=0.1)	= at <48h	
			-		Mild HIE				

(Haiju, Suyuan et al. 2008) [45]	Cord blood	Nucleated red blood cells	May-Grunwald-Giemsa stain (cytometer)	Moderate/severe HIE	Increased levels compared to mild HIE ($p<0.01$)	↑ at 0h

Table 6 – Summary of findings of biomarkers present in whole-blood.

Reference	Sample type	Name	Code	Technique	Group	Collection time	Results	Summary	Observations
**Proteins**									
(Maggiotto, Sondhi et al. 2019) [29]	Whole blood	GLUT1	P11166	ELISA	Control	6h-76h	Increased levels in pre-TH compared to control groups (p=0.0057)	↑ at pre-TH	GLUT1 was quantified in red blood cells Decreased levels comparing pre-TH and post-TH (p=0.05)
					HIE	Pre-TH; during TH; rewarming; pos-TH			
		GLUT3	P11169		Control	6h-76h	No significant differences	= on all timepoints	GLUT3 was quantified in white blood cells
					HIE	Pre-TH; during TH; rewarming; pos-TH			
**Metabolites**									
(Bersani, Ferrari et al. 2019) [44]	Whole blood	Creatinine	HMDB0000562	-	Moderate HIE (with hypothermia)	At birth	No statistical differences (p>0.05)	= on all groups	Data is also available for groups that were not treated with TH
					Severe HIE (with hypothermia)				
		Urea	HMDB0000294	-	Moderate HIE (with hypothermia)	At birth	No statistical differences (p>0.05)	= on all groups	
					Severe HIE (with hypothermia)				
(Jain, Pagano et al. 2017) [27]	Whole-blood	Lactate	HMDB0000190	-	HIE (with MRI score 0 or 1)	At admission	No statistical differences (p=0.21)	= on all groups	
					HIE (with MRI score 2)				
					HIE (with MRI score 3)				
**RNA**									
(Balada, Tebe et al. 2020) [20]	Whole-blood	CCR5	GC03P046383	RT-qPCR	Control	6h, 12h, 24h, 48h, 72h, 96h	Decreased levels compared to controls (p<0.001)	↓ on all timepoints	
					HIE				
					Moderate/severe HIE		Lower levels compared to mild HIE (p=0.003)		
		PPARG	GC03P012287		Control	6h, 12h, 24h, 48h, 72h, 96h	Increased levels compared to controls (p=0.002)	↑ on all timepoints	Faster decrease on mild HIE compared to moderate/severe HIE (p = 0.074)
					HIE				
		MMP9	GC20P046008		Control	6h, 12h, 24h, 48h, 72h, 96h	Increased levels compared to controls (p<0.001); gradual decrease over time	↑ on all timepoints	
					HIE				
					Mild HIE	6h, 12h, 24h, 48h, 72h, 96h	Faster decrease on mild infants compared to moderate/severe (p=0.007)		
		IL-8	GC04P073740		Control	6h, 12h, 24h, 48h, 72h, 96h	Increased levels compared to controls (p=0.004); gradual decrease over time	↑ on all timepoints	Increased levels at 6h-12h correlated with negatives outcomes
					HIE				

					Severe HIE	6h, 12h	Increased levels compared to mild HIE (p<0.01) and moderate HIE (p<0.01)	
		HSPA1A	GC06P070160		Control			
					HIE	6h, 12h, 24h, 48h, 72h, 96h	Increased levels compared to controls (p=0.005); gradual decrease over time	↑ on all timepoints
					Severe HIE	6h, 12h	Increased levels compared to mild HIE (p<0.001) and moderate HIE (p<0.001)	
		TLR8	GC0XP012924		Control			
					HIE	6h, 12h, 24h, 48h, 72h, 96h	Increased levels compared to controls (p=0.003); gradual decrease over time	↑ on all timepoints
**miRNA**								
(Ponnusamy, Kapellou et al. 2016) [31]	EDTA-blood	RNU6B	GC10P013220	TaqMan miRNA assay	HIE	18h-19h	No comparisons between groups were performed	
		Let7b	GC22P046119					
		miR-21	GC17P059841					
**Number of cells**								
(Saito, Shibasaki et al. 2016) [33]	Whole-blood	White-blood cells	-	Flow cytometry	HIE	24h, 48h, 72h, 96h, 120h, 144h, 168h	Negative correlation with CRP levels	
		Neutrophils	-				No statistical differences were found	
		Platelet	-					

Table 7 – Summary of findings of biomarkers present in dried-blood spots.

Reference	Sample type	Name	Code	Technique	Group	Collection time	Results	Summary	Observations
**Proteins**									
(Massaro, Wu et al. 2019)[32]	DBS	S100B	P04271	ELISA	HIE	<24h, 48h, 72h, 120h			
		IL-1β	P01584	V-PLEX proinflammatory panel					
		Erythropoietin	P01588	Human EPO base kit					
		IL-6	P05231	V-PLEX proinflammatory panel	HIE (favorable outcome)<br>HIE (unfavorable outcome)		No relationship with death, severe brain injury (p>0.05) or neurodevelopment impairment (p>0.05)	= comparing the favorable and unfavorable outcome	
		IL-8	P10145		HIE (favorable outcome)<br>HIE (unfavorable outcome)				
		TNF-α	P01375		HIE (favorable outcome)<br>HIE (unfavorable outcome)				
**Metabolite**									
(Lopez-Suarez, Concheiro-Guisan et al. 2019)[21]	DBS	C2	HMDB0240773	ESI-MS/MS	Control<br>HIE	72h	Decreased levels compared to controls (p=0.000)	↓ at 72h	
		C3:1	HMDB0000824		HIE (favorable outcome)		Increased levels compared to HIE to unfavorable outcome (p=0.001)	↑ at 72h	
		C3DC	HMDB0002095		HIE (favorable outcome)		Increased levels compared to HIE to unfavorable outcome (p=0.005)	↑ at 72h	
		C4	HMDB0002013		Control<br>HIE		Increased levels compared to controls (p=0.000)	↑ at 72h	
		C4-OH	HMDB0013127		Control<br>HIE		Decreased levels compared to controls (p=0.005)	↓ at 72h	
					HIE (favorable outcome)		Increased levels compared to HIE to unfavorable outcome (p=0.01)	↑ at 72h	
		C5	HMDB0000378; HMDB0000688		Control<br>HIE		Increased levels compared to controls (p=0.000)	↑ at 72h	
		C5:1	HMDB0002366		Control<br>HIE		Increased levels compared to controls (p=0.04)	↑ at 72h	
		DC5	HMDB0013130		HIE (favorable outcome)		Increased levels compared to HIE to unfavorable outcome (p=0.004)	↑ at 72h	
		C6	HMDB0000756		Control<br>HIE		Increased levels compared to controls (p=0.000)	↑ at 72h	
		C6-OH	HMDB0013131		Control<br>HIE		Increased levels compared to controls (p=0.001)	↑ at 72h	

		HIE (unfavorable outcome)	Increased levels compared to HIE to favorable outcome (p=0.031)	↑ at 72h
C8	HMDB0000791	Control<br>HIE	Increased levels compared to controls (p=0.023)	↑ at 72h
		HIE (unfavorable outcome)	Increased levels compared to HIE to favorable outcome (p=0.001)	↑ at 72h
C8:1		HIE (unfavorable outcome)	Increased levels compared to HIE to favorable outcome (p=0.011)	↑ at 72h
C10	HMDB0000651	HIE (unfavorable outcome)	Increased levels compared to HIE to favorable outcome (p=0.012)	↑ at 72h
C10:1		HIE (unfavorable outcome)	Increased levels compared to HIE to favorable outcome (p=0.023)	↑ at 72h
C10:2	HMDB0241102	HIE (unfavorable outcome)	Increased levels compared to HIE to favorable outcome (p=0.009)	↑ at 72h
C12	HMDB0002250	Control<br>HIE	Decreased levels compared to controls (p=0.000)	↓ at 72h
C14	HMDB0005066	Control<br>HIE	Decreased levels compared to controls (p=0.000)	↓ at 72h
C14:1		Control<br>HIE	Decreased levels compared to controls (p=0.000)	↓ at 72h
C14:2		Control<br>HIE	Decreased levels compared to controls (p=0.000)	↓ at 72h
C14:1-OH	HMDB0253296	HIE (unfavorable outcome)	Increased levels compared to HIE to favorable outcome (p=0.032)	↑ at 72h
C14-OH		HIE (unfavorable outcome)	Increased levels compared to HIE to favorable outcome (p=0.004)	↑ at 72h
C16	HMDB0000222	Control<br>HIE	Decreased levels compared to controls (p=0.000)	↓ at 72h
C16:1		Control<br>HIE	Decreased levels compared to controls (p=0.000)	↓ at 72h
C16-OH	HMDB0241520	HIE (unfavorable outcome)	Increased levels compared to HIE to favorable outcome (p=0.048)	↑ at 72h
C18	HMDB0000848	Control<br>HIE	Decreased levels compared to controls (p=0.000)	↓ at 72h
		HIE (unfavorable outcome)	Increased levels compared to HIE to favorable outcome (p=0.03)	↑ at 72h
C18:1	HMDB0255949	Control<br>HIE	Decreased levels compared to controls (p=0.000)	↓ at 72h
C18-OH	HMDB0241520	Control<br>HIE	Decreased levels compared to controls (p=0.028)	↓ at 72h
		HIE (unfavorable outcome)	Increased levels compared to HIE to favorable outcome (p=0.03)	↑ at 72h
C18:1-OH	HMDB0255949<br>)	HIE (favorable outcome)	Increased levels compared to HIE to unfavorable outcome (p=0.001)	↑ at 72h

		MeGlut	HMDB0254648		HIE (favorable outcome)		Increased levels compared to HIE to unfavorable outcome (p=0.002)	↑ at 72h
		Free carnitine	-		Control<br>HIE		No significant differences	= on both groups
		Free/total carnitine ratio	-		Control<br>HIE		No significant differences	= on both groups
**miRNA**								
(Ponnusamy, Kapellou et al. 2016) [31]	DBS	Let7b	GC22P046119	TaqMan miRNA assay	HIE (favorable outcome)<br>HIE (unfavorable outcome)	18h-19h	No significant differences	= on both groups
		miR-21	GC17P059841		HIE (favorable outcome)<br>HIE (unfavorable outcome)		No significant differences	= on both groups
		miR-29b	GC07M130877; GC01M207806		HIE (favorable outcome)<br>HIE (unfavorable outcome)		No significant differences	= on both groups
		miR-124	GC08M009903; GC20P063181; GC08P064379		HIE (favorable outcome)<br>HIE (unfavorable outcome)		No significant differences	= on both groups
		miR-155	GC21P025573		HIE (favorable outcome)<br>HIE (unfavorable outcome)		No significant differences	= on both groups
		RNU6B	GC10P013220		HIE		No comparisons between groups were performed	
